# Insight into the distinctive paradigm of Human Cytomegalovirus associated intrahepatic and extrahepatic cholestasis in neonates

**DOI:** 10.1038/s41598-020-73009-z

**Published:** 2020-09-28

**Authors:** Aroni Chatterjee, Sumit Mukherjee, Biswanath Basu, Debsopan Roy, Rivu Basu, Hiya Ghosh, Lopamudra Mishra, Mala Bhattacharya, Nilanjan Chakraborty

**Affiliations:** 1 Virus Research Laboratory, ICMR-National Institute of Cholera and Enteric Diseases, GB4, ID & BG Hospital Campus, Dr. S.C Banerjee Road, Beliaghata, Kolkata, West Bengal 700010 India; 2 Azrieli Faculty of Medicine, Bar-Ilan University, Safed, Israel; 3 Department of Paediatrics, N. R. S. Medical College & Hospital, Kolkata, 700014 India; 4 Department of Community Medicine, R. G. Kar Medical College and Hospital, Kolkata, 700004 India; 5 Department of Endocrinology and Metabolism, IPGMER & SSKM Hospital, Kolkata, 700020 India; 6 Department of Paediatrics, Dr. B. C. Roy Post Graduate Institute of Paediatric Sciences, Kolkata, 700054 India

**Keywords:** Diseases, Gastroenterology, Medical research, Risk factors

## Abstract

Human Cytomegalovirus has been implicated as a probable cause for the development of hepatic cholestasis among neonates. Our study tried to ascertain the exact demographic, biochemical and immunological markers to differentially diagnose patients with HCMV associated intrahepatic and extrahepatic cholestasis and also decipher the phylogenetic variability among the viral strains infecting the two groups. A total of 110 neonates collected over a span of 2 years were selected for the study classified into four different groups based on the presence of hepatic cholestasis and active HCMV infection. Our analysis predicted that total Cholesterol, GGT, ALP and TNFα were the only significant biological markers with exact cut-off scores, capable of distinguishing between HCMV associated intrahepatic and extrahepatic cholestasis. We confirmed that in patients belonging to both of these groups, the inflammasome is activated and the extent of this activation is more or less same except for the initial activators NLRP3 and AIM2 respectively. When we performed two separate phylogenetic analyses with HCMV gM and gN gene sequences, we found that in both cases the sequences from the IHC and EHC groups formed almost separate phylogenetic clusters. Our study has shown that the HCMV clinical strains infecting at intrahepatic and extrahepatic sites are phylogenetically segregated as distinct clusters. These two separate groups show different physiological as well as immunological modulations while infecting a similar host.

## Introduction

Neonatal hepatic cholestasis is broadly defined to be a medical condition characterized by a significant reduction in the secretion or flow of bile contemplating with the appearance of conjugated hyperbillirubinemia occurring in newborns during the very first few months of life^1,2^. Hepatic cholestasis can be classified into intrahepatic, involving mainly the liver parenchymal cells or extrahepatic, relating to any excretory block outside of the liver^3,4^. Human cytomegalovirus (HCMV), a ubiquitous opportunistic β-herpesvirus has been occasionally implicated in the development of hepatic cholestasis among neonates^5^. Many studies have provided evidences linking the association between active HCMV infection and development of hepatic cholestasis in different individuals^6^. Inflammatory response pathways play a major role in the etiology of hepatic cholestasis and since HCMV infections in neonates triggers a major immune response it is likely that this causes or aggravates the development of hepatic cholestasis^7,8^. During cholestasis, excessive inflammatory responses can promote secretion of more proinflammatory cytokines, to amplify inflammatory damage and liver fibrosis^9^. Moreover, these responses can subsequently activate the inflammasome complex causing extensive liver fibrosis and cirrhosis^10,11^. An important route of innate immunity, the first line of defence against HCMV relies on the multimeric protein complex called inflammasome^12,13^. This complex is formed by different sensor and adaptor proteins which together converts the inactive pro-caspase-1 into its active protease form. The caspase-1 in its turn catalyzes the maturation of pro-inflammatory cytokines IL-1β and IL-18^14,15^. The major part of what we currently know about inflammasome modulation during cytomegalovirus (CMV) infection has been derived from experiments with mouse CMV (MCMV), even though there have been few reports showing inflammasome activation in HCMV-infected PBMCs of infected individuals^16,17^. HCMV expresses a huge number of surface glycoproteins among which the gM/gN glycoprotein complex (gCII complex) is the most abundant complex on the virion surface and has been reported to play key roles during attachment to host cells, likely by mediating interactions with heparan sulfate proteoglycans on the cell surface thereby facilitating virus entry into host cells^18,19^. The gM/gN protein complex have shown to elicit a huge virus-neutralizing antibody response in humans infected with HCMV and has also been identified as an additional major antigen for the humoral immune response against HCMV^20,21^. Early recognition of the type and cause of neonatal cholestasis is essential for optimal prognosis and successful treatment. By analysing the different associated physiologicaland immunological parameters we will be able to formulate a more advanced defining criterion for predicting and monitoring cases of HCMV associated intrahepatic and extrahepatic cholestasis among neonates thereby generating a better therapeutic advantage. Simultaneously we would also decipher the pattern of variability in the phylogenetic lineage of the HCMV clinical strains inducing intrahepatic and extrahepatic cholestasis with respect to specific viral genes.

## Results

### Distinctive profiling of patients with HCMV associated hepatic cholestasis based on different baselineparameters

#### Patient’s comparative profile for demographic and physical parameters

Out of all the demographic and physical parameters tested only abdominal circumference and head circumference values showed significant variations among group 2 and group 3 patients with respect to group 1. Mean value for abdominal circumference was similar and significantly high in case of patients with hepatic cholestasis i.e. Group 1 and group 3, compared to patients without hepatic cholestasis i.e. Group 2 and group 4. There happens to be a significant difference in mean abdominal circumference value for group 2 when compared to group 1. Reduced head circumference was observed in case of patients with active HCMV infection i.e. both group 1 and group 2 compared to patients without HCMV infection i.e. group 3 and group 4. The detailed comparative analysis has been provided in Table 1.

**Table 1:** A comparative analysis of the significant (p ≤ 0.05) demographic and biochemical parameters of the infants measured at the time of admission, differentiating Group 1 (N = 35) from Group 2 (N = 25), Group 3 (N = 25) and Group 4 (N = 25).

Parameters	Mean ± SD	Significance (P value)	95% Confidence interval	
			Lower	Upper
**Head circumference (Cms)**				
Group 1	33.15 ± 1.64	Constant		
Group 2	33.04 ± 1.47	1.000	− 1.377	1.6
Group 3	35.62 ± 2.36	< 0.001	− 3.991	− 1.013
Group 4	35.85 ± 1.73	0.002	− 4.051	− 1.346
**Abdominal circumference (Cms)**				
Group 1	38.63 ± 2.68	Constant		
Group 2	35.52 ± 5.68	0.048	− 0.0025	6.226
Group 3	38.82 ± 4.23	1.000	− 3.302	2.92
Group 4	35.13 ± 3.76	0.008	0.6682	6.32
**Albumin (mg/dL)**				
Group 1	3.8 + 0.89	Constant		
Group 2	1.85 + 0.44	< 0.001	1.347	2.53
Group 3	3.54 + 0.79	1.000	− 0.3438	0.8417
Group 4	1.87 + 0.35	< 0.001	1.38	2.46
**D. Billirubin (mg/dL)**				
Group 1	9.8 ± 0.97	Constant		
Group 2	2.65 ± 0.53	< 0.001	5.48	6.82
Group 3	9.65 ± 0.82	1.000	− 0.5166	0.8268
Group 4	2.55 ± 0.59	< 0.001	4.63	5.85
**SGPT (IU/L)**				
Group 1	200.7 ± 20.1	Constant		
Group 2	54.2 ± 8.94	< 0.001	131.86	161.33
Group 3	177.7 ± 25.29	< 0.001	8.32	37.79
Group 4	50.4 ± 9.03	< 0.001	137.01	163.77
**SGOT (IU/L)**				
Group 1	247.4 ± 39	Constant		
Group 2	44.78 ± 4.07	< 0.001	179.33	226.002
Group 3	197.9 + 27.18	< 0.001	26.173	72.843
Group 4	41.35 ± 6.82	< 0.001	184.896	227.286
**Alkaline phosphatase (IU/L)**				
Group 1	761.3 ± 36.3	Constant		
Group 2	230.7 ± 61.7	< 0.001	485.13	577.339
Group 3	545.16 ± 89.9	< 0.001	170.05	262.32
Group 4	234.1 ± 43.42	< 0.001	485.32	569.123
**Gamma glutamyl transferase (IU/L)**				
Group 1	87.7 + 6.36	Constant		
Group 2	42.01 + 6.5	< 0.001	40.539	50.835
Group 3	63.4 + 6.11	< 0.001	19.082	29.378
Group 4	37.5 + 5.55	< 0.001	45.482	54.833
**Globulin (gm/dL)**				
Group 1	2.97 ± 0.58	Constant		
Group 2	2.69 ± 0.47	0.544	− 0.1586	0.7061
Group 3	2.5 ± 0.39	0.025	0.0387	0.9034
Group 4	2.35 ± 0.49	< 0.001	0.2240	1.0094
**Cholesterol (mg/dL)**				
Group 1	259.07 + 76.34	Constant		
Group 2	195.8 + 16.49	0.001	20.351	106.184
Group 3	210.38 + 27.6	0.018	5.771	91.604
Group 4	121.11 + 8.96	< 0.001	98.979	176.94
**TGL (mg/dL)**				
Group 1	244.9 ± 35.6	Constant		
Group 2	184.4 ± 9.69	< 0.001	48.903	92.175
Group 3	248.6 ± 21.1	0.986	− 25.377	17.895
Group 4	181.1 ± 14.5	< 0.001	44.104	83.408
**LDL (mg/dL)**				
Group 1	191.2 ± 14.9	Constant		
Group 2	131.3 ± 14.6	< 0.001	48.53	71.29
Group 3	182.2 ± 14.9	0.203	− 2.29	20.46
Group 4	103.5 ± 8.5	< 0.001	77.41	98.08
**Phosphate (mg/dL)**				
Group 1	5.15 ± 0.58	Constant		
Group 2	5.8 ± 0.9	0.007	− 1.218	− 0.1313
Group 3	4.8 ± 0.57	0.789	− 0.2375	− 0.8501
Group 4	5.9 ± 0.58	0.001	− 1.339	− 0.3513
**Potassium (mEq/L)**				
Group 1	1.54 ± 0.43	Constant		
Group 2	2.17 ± 0.68	0.016	− 1.1779	− 0.0795
Group 3	3.2 ± 0.9	< 0.001	− 2.2092	− 1.1108
Group 4	3.36 ± 0.76	< 0.001	− 2.3163	− 1.3187
**Calcium (mg/dL)**				
Group 1	7.9 ± 1.6	Constant		
Group 2	6.24 ± 0.52	< 0.001	0.6474	− 2.6635
Group 3	6.39 ± 0.88	0.001	0.4960	2.5122
Group 4	5.5 ± 0.85	< 0.001	1.4813	3.3125
**Lactate (mmol/L)**				
Group 1	3.59 ± 0.64	Constant		
Group 2	2.9 ± 0.43	0.001	0.2386	1.1369
Group 3	3.02 ± 0.38	0.006	0.1172	1.015
Group 4	2.2 ± 0.49	< 0.001	0.9653	1.7812
**Sodium (mEq/L)**				
Group 1	133.22 + 21.93	Constant		
Group 2	108.69 + 12.7	< 0.001	10.11	38.95
Group 3	100.45 + 9.16	< 0.001	18.35	47.19
Group 4	101.44 + 15.18	< 0.001	18.68	44.88
**INR**				
Group 1	2.08 ± 0.71	Constant		
Group 2	1.84 ± 0.53	1.000	− 0.2464	0.7201
Group 3	1.55 ± 0.51	0.026	0.0403	1.0068
Group 4	1.25 ± 0.32	< 0.001	0.3919	1.269

Mean + SD values were calculated and one way ANOVA was performed using Bonferroni method (Post-hoc analysis) for comparing the mean values of each group with respect to that of Group 1.

#### Patient’s comparative profile for serum biochemical markers

Out of all the serum biochemical markers tested at the time of admission only nine (SGOT, SGPT, ALP, GGT, Cholesterol, sodium, potassium, calcium and lactate) showed statistically significant differences between group 1 and both groups 2 and 3. These parameters were chosen as critical markers of HCMV associated hepatic cholestasis as they were able to clearly differentiate group 1 from all other groups. Apart for the critical parameters chosen above, the mean values for Albumin, Billirubin, TGL, LDL and phosphate showed significant differences between group 2 and group 1 only. The values for these 5 parameters were quite similar in case of group 1 and group 3 suggesting that the variation in the concentration of these parameters were governed by the presence of hepatic cholestasis alone in case of both groups. Globulin and INR were found to have significant differences between group 1 and group 3 only and similar values in case of group 1 and group 2 indicating that these variations were most likely due to active HCMV infection in both groups. The detailed comparative analysis has been provided in Table 1.

#### Patient’s comparative profile for serum immunological markers

To test the variable expression pattern of some selective inflammatory markers among the patients belonging to the four groups, ELISA was performed from the serum samples. Among the markers tested only TNFα, IL1β, IL1Ra, IL18 and CRP showed statistically significant variations among groups 2 and 3 with respect to group 1 and were hence selected as critical markers of HCMV associated hepatic cholestasis. Mean concentrations of TNFα, IL1β, IL18, and CRP were significantly elevated in group 1 patients compared to the other groups whereas that of IL1Ra was significantly reduced compared to others. In patients with active HCMV infection i.e. group 1 and group 2, mean concentrations of IFNγ, IL10 and IL8 were found to be quite similar. No variation was found in the concentrations of TGFβ and IL6 among the patient groups. Detailed comparative analyses for each group have been provided in Table 2.

**Table 2:** A comparative analysis of all the serum immunological markers measured in the infants at the time of presentation, differentiating between Group 1 (N = 35) from Group 2 (N = 25), Group 3 (N = 25) and Group 4 (N = 25).

Inflammatory markers	Mean ± SD	Significance	95% Confidence interval	
			Lower	Upper
**TNFα (pg/mL)**				
Group 1	185.4 ± 40.24	Constant		
Group 2	130.76 ± 9.91	0.000	30.96	78.34
Group 3	121.94 ± 12.9	0.000	39.78	87.16
Group 4	49.44 ± 18.42	0.000	114.45	157.48
**IFNγ (pg/mL)**				
Group 1	19.14 ± 5.5	Constant		
Group 2	19.52 ± 7.59	1.000	− 4.4368	3.67
Group 3	4.56 ± 2.06	0.000	10.52	18.62
Group 4	3.56 ± 0.78	0.000	11.88	19.25
**IL1β (pg/mL)**				
Group 1	149.5 ± 17.87	Constant		
Group 2	61.5 ± 8.35	0.000	76.611	99.394
Group 3	37.26 ± 13.19	0.000	100.858	123.64
Group 4	32.6 ± 6.53	0.000	106.545	127.239
**IL10 (pg/mL)**				
Group 1	17.88 ± 6.49	Constant		
Group 2	17.7 ± 2.85	1.000	− 5.736	6.079
Group 3	34.9 ± 8.27	0.000	− 22.996	− 11.18
Group 4	34.95 ± 9.02	0.000	− 22.439	− 11.707
**TGFb (ng/mL)**				
Group 1	3.58 + 0.62	Constant		
Group 2	3.51 + 0.73	1.000	− 0.5455	0.6834
Group 3	3.58 + 0.87	1.000	− 0.6148	0.6140
Group 4	3.5 + 0.82	1.000	− 0.4753	0.6409
**IL6 (pg/mL)**				
Group 1	39.07 ± 6.15	Constant		
Group 2	40.38 ± 12.7	1.000	− 8.160	5.551
Group 3	42.7 ± 7.35	0.926	− 10.50	3.211
Group 4	39.9 ± 7.77	1.000	− 7.061	5.392
**IL1Ra (pg/mL)**				
Group 1	141 ± 9.88	Constant		
Group 2	667.14 ± 59.9	0.000	− 567.05	− 485.224
Group 3	622.86 ± 77.7	0.000	− 522.77	− 440.944
Group 4	651.2 ± 54.5	0.000	− 547.369	− 473.047
**IL18 (pg/mL)**				
Group 1	490.29 ± 33.35	Constant		
Group 2	248.36 ± 69.79	0.00	205.154	278.714
Group 3	269.14 ± 54.14	0.00	184.374	257.934
Group 4	193.92 ± 23.19	0.00	262.962	329.777
**IL8 (pg/mL)**				
Group 1	25.13 ± 4.81	Constant		
Group 2	23.33 ± 7.4	1.000	− 2.615	6.207
Group 3	12.09 ± 5.45	0.000	8.622	17.44
Group 4	11.34 ± 3.92	0.000	9.782	17.796
**CRP (mg/L)**				
Group 1	19.04 ± 1.57	Constant		
Group 2	10.92 ± 3.15	0.000	6.398	9.829
Group 3	2.46 ± 0.73	0.000	14.856	18.287
Group 4	3.39 ± 2.42	0.000	14.085	17.201

Mean ± SD values were calculated and one way ANOVA was performed using Bonferroni method for comparison with respect to Group 1.

### Distinctive profiling of patients with HCMV associated intrahepatic cholestasis (IHC) and extrahepatic cholestasis (EHC) based on selectedmarkers

We intended to test whether the significant parameters that we have selected previously can help us to distinctively classify between the HCMV associated intrahepatic and extrahepatic cases. For this purpose univariate binary logistic regression was performed using the selected parameters (nine serum biochemical and five inflammatory). 31 patients from group 1 with either intra (N = 12) or extra hepatic cholestasis (N = 19) were chosen as the target population for analysis. The analysis revealed that out of the parameters tested; only seven parameters like abdominal circumference, GGT, ALP, calcium, Cholesterol, sodium and TNF α could significantly predict the occurrence of intrahepatic cholestasis among the group 1 patients with either intra or extrahepatic cholestasis. The mean concentrations for GGT, total cholesterol, ALP and TNF α were found to be higher in case of patients with IHC whereas the mean concentrations for sodium and calcium were higher in case of patients with EHC. A detailed analysis has been provided in Table 3.

**Table 3:** Binary logistic regression analysis of the selected significant biochemical and immunological factors to ascertain their role in significantly comparing between HCMV induced intrahepatic (IHC) and extrahepatic (EHC)cases.

Parameters	Mean ± SD	Significance	Beta coefficient	95% CI	
				Lower	Upper
**SGPT**					
IHC	202.68 ± 24.5	0.38	0.982	0.945	1.022
EHC	196.5 ± 14.75				
**SGOT**					
IHC	245.12 ± 39.67	0.641	1.005	0.985	1.025
EHC	251.52 ± 37.35				
**GGT**					
IHC	92.94 ± 5.7	0.009	0.769	0.631	0.937
EHC	84.09 ± 7.11				
**Potassium**					
IHC	1.55 ± 0.4	0.849	0.851	0.162	4.48
EHC	1.52 ± 0.47				
**Calcium**					
IHC	6.07 ± 1.22	0.023	29.868	1.599	557.74
EHC	8.82 ± 0.78				
**Total cholesterol**					
IHC	344.9 ± 46.3	0.041	0.874	0.76	1.006
EHC	214.9 ± 46.36				
**Lactate**					
IHC	3.72 ± 0.57	0.273	0.49	0.137	1.752
EHC	3.45 ± 0.72				
**ALP**					
IHC	826.5 ± 81.82	0.024	0.967	0.939	0.996
EHC	718.06 ± 66.7				
**Sodium**					
IHC	113.3 ± 12.65	0.003	1.133	1.045	1.23
EHC	148.03 ± 16.83				
**TNFα**					
IHC	251.67 ± 42.98	0.002	0.953	0.924	0.983
EHC	141.97 ± 36.87				
**IL1β**					
IHC	148.2 ± 28.89	0.756	1.006	0.968	1.046
EHC	150.35 ± 9.44				
**IL1Rα**					
IHC	138.28 ± 8.93	0.16	1.059	0.978	1.148
EHC	143.43 ± 10.10				
**IL18**					
IHC	481.3 ± 41.54	0.283	1.012	0.990	1.034
EHC	495.4 ± 30.38				
**CRP**					
IHC	18.96 ± 1.18	0.725	1.087	0.684	1.726
EHC	19.16 ± 1.85				

### Determination of cut-off values for significant parameters in predicting intrahepatic or extrahepatic cholestasis

**Figure 1:**
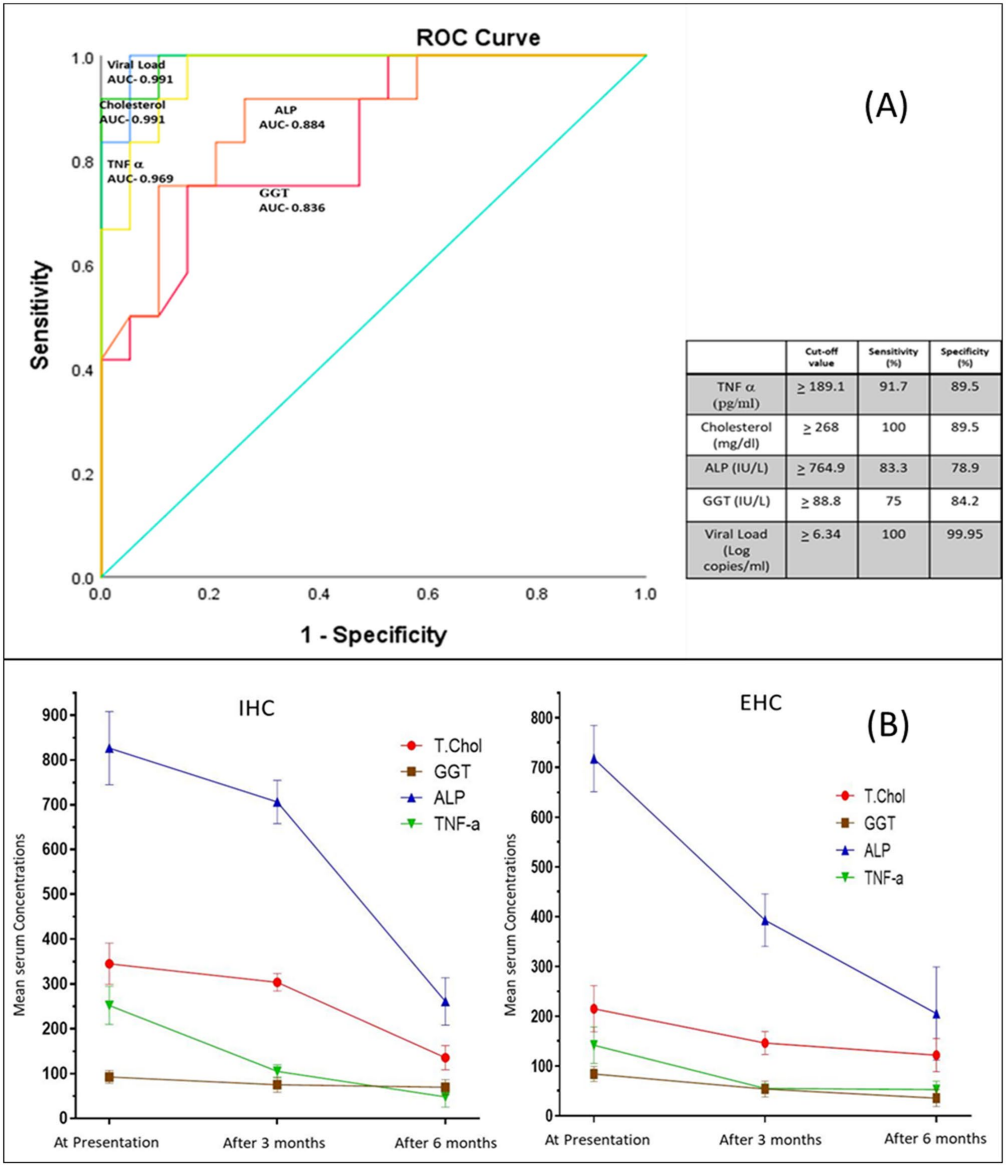
(**A**) ROC curves for the significant biomarkers defining HCMV associated IHC and HCMV blood viral load with best predictive accuracy (Area under the Curve > 0.8). (**B**) A comparative follow-up analysis of the four critical markers with highest predictive accuracy differentiating between HCMV associated intrahepatic cholestasis (IHC) and extrahepatic cholestasis (EHC).

Receiver-operating characteristic (ROC) curves of the selected models were derived for all the chosen critical biomarkers. The curves were used to evaluate the individual cut-off values prioritizing both highest sensitivity and specificity. Of the seven predictive biomarkers associated with HCMV induced IHC in univariate models, Cholesterol, TNFα, ALP and GGT showed the highest predictive power [Area under the curve (AUC) > 0.8], where Cholesterol and TNFα demonstrated best accuracy as single biomarkers (AUC = 0.991 [95% CI 0.968–1.00] and AUC = 0.969 [95% CI 0.919–1.00] respectively). ALP and GGT also showed good accuracy scores with AUCs of 0.884 and 0.836 respectively (Fig. 1A).

HCMV blood viral load was determined separately for group 1 patients i.e. those with HCMV associated hepatic cholestasis. Mean value of viral log copies/mL was found to be higher in case of patients with IHC (7.53 ± 0.97) than those with EHC (5.66 ± 0.48). Receiver-operating characteristic (ROC) curve for viral load was found to have a very high predictive accuracy (AUC-0.991 [95% CI 0.968–1.00]) (Fig. 1A).

### Follow-up analysis

Detailed follow-up analysis of all the parameters for all patients were done to monitor the variation in the state of expression of these parameters after 3 and 6 months of initial disease presentation (Data not shown). The Follow up analysis for the four previously described critical biomarkers (Total Cholesterol, ALP, GGT and TNFα), capable of differentiating between the HCMV associated IHC and EHC patient groups has been depicted in Fig. 1B.

Follow up after 3 months and 6 months revealed that the mean values of some of these parameters which were significant during the time of disease presentation still remained significantly different among the HCMV associated IHC and EHC patient groups. The parameters, total Cholesterol (P ≤ 0.001), GGT (P = 0.001), ALP (P ≤ 0.001) and TNFα (P ≤ 0.001) remained significant till 3 months but not at 6 months. The mean values of all these four biomarkers in case of IHC patients (Mean_T.chol_-303.25 mg/dL ± 19.38, Mean_GGT_-74.85 IU/L ± 16.9, Mean_ALP_-706.22 IU/L ± 48.65 and Mean_TNFα-_104.97 pg/mL ± 14.8) remained significantly high compared to the EHC patients (Mean_T.chol_-146.29 mg/dL ± 23.1, Mean_GGT_-53.78 IU/L ± 15.98, Mean_ALP_-392.92 IU/L ± 52.4 and Mean_TNFa_-55.22 pg/mL ± 8.19) even after 3 months. Only GGT (P ≤ 0.001) remained significant till 6 months of presentation with mean value of 69.47 IU/L ± 16.84 for IHC patients and 35.56 IU/L + 16.74 for EHC patients.

### Assessment of all the accessory clinical conditions differentiating between IHC and EHC

Next we tried to categorize the different accessory clinical conditions present among the patients with HCMV associated intra and extra hepatic cholestasis. Infantile cholangiopathy was the most common manifestation present in the neonates with IHC (83.33% cases), followed by hepatomegaly among 75% of the cases and hyperplasia among 66.66% of the cases. In case of neonates with HCMV associated EHC, biliary atresia was the most common accessory manifestation present (78.9% cases), followed by hepatomegaly among 57.8% patients, biliary dyskinesia and fecal acholia both among 47.36% patients. The detailed comparative assessment of all the accessory clinical conditions present has been depicted in Supplementary Fig. 1.

### Differential mRNA expression of immunological markers in PBMCs of patients with HCMV associated hepatic cholestasis

High secretory protein level of IL1β (149.5 pg/mL ± 17.87) and IL 18 (490.29 pg/mL ± 33.35) measured using ELISA from patients’ blood serum immediately after admission indicated towards a probable activation of inflammasome pathway among the group 1 patients. To confirm our assumption, relative mRNA expression ratio of four different inflammasome components and some other cytokines/chemokines in the PBMCs of the patients were assessed and compared between the four groups. Four biomarkers (IL1β, Caspase1, ASC and NLRP3) for predicting activated inflammasome pathway were chosen along with TNFα, MCP1, MIP1α, TGFβ, IFNγ, IL2, RIG1 and RANTES.

**Figure 2:**
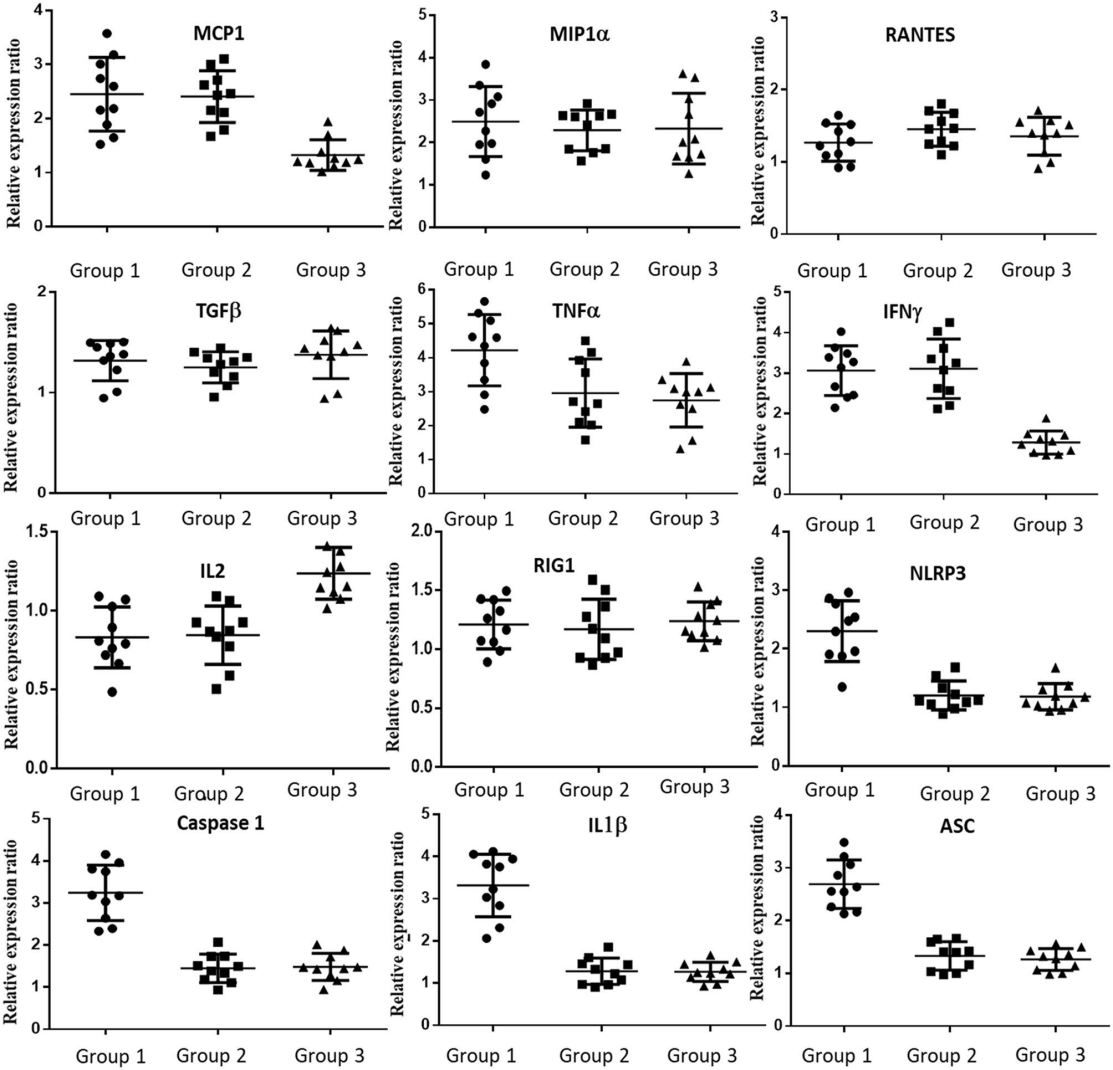
Differential mRNA expression of some selected chemokines, cytokines and inflammasome pathway components in PBMCs from patients belonging to the three different groups (N = 10). Their relative mRNA expression ratio with respect to group 4 (control group) in terms of fold change (2^−ΔΔCt^) were estimated by quantitative real time PCR. GAPDH mRNA served as internal control. (+ 1 value in Y axis was considered to be the baseline of control, with values greater than 1 suggesting positive fold change and values below 1 upto 0 as negative fold change).

The relative expression ratio of MCP1 and IFNγ were found to be similar in case of group 1 and group 2 and significantly higher when compared to group 3. The fold change in mRNA expression of MCP1 and IFNγ were positively significant in both the groups compared to control. This suggest that active HCMV infection was directly associated with their increased expression. There was no significant change in the mRNA expression pattern of MIP1α. TGFβ, RIG1 and RANTES among the groups as well as when compared to control. Expression of TNFα was found to be significantly high in case of group 1 patients with respect to group 2 and group 3 and was found to be significantly increased in all the three groups when compared to control. This was in accordance with our ELISA results. Expression of IL2 significantly decreased in case of group 1 and group 2 patients i.e. those with active HCMV infection than group 3 patients. The mRNAs of all the four chosen components of inflammasome pathway were found to be expressed at a significantly higher level in case of patients with HCMV associated hepatic cholestasis i.e. Group 1 compared to all other groups. The positive (increased) fold change of these components suggested that only in group 1 patients there was a probable activation of the inflammasome pathway. The expression changes of these four markers in case of group 2 and group 3 were almost negligible compared to the control group. Figure 2 provides a clear representation of our findings and the detailed statistical analysis has been provided in the Supplementary Table 3.

### Differential activation of inflammosome pathway in PBMCs of patients with HCMV associated intrahepatic and extrahepatic cholestasis

As our previous experiments confirmed that the inflammasome pathway is indeed activated in the PBMCs of group 1 patients, we intended to decipher whether there exists any difference in the process of activation between those with intrahepatic and extrahepatic cholestasis. To test our assumptions we studied the mRNA expression ratio of nine inflammatory pathway components in the PBMCs of patients with intrahepatic and extrahepatic cholestasis (12 patients for each group).

**Figure 3:**
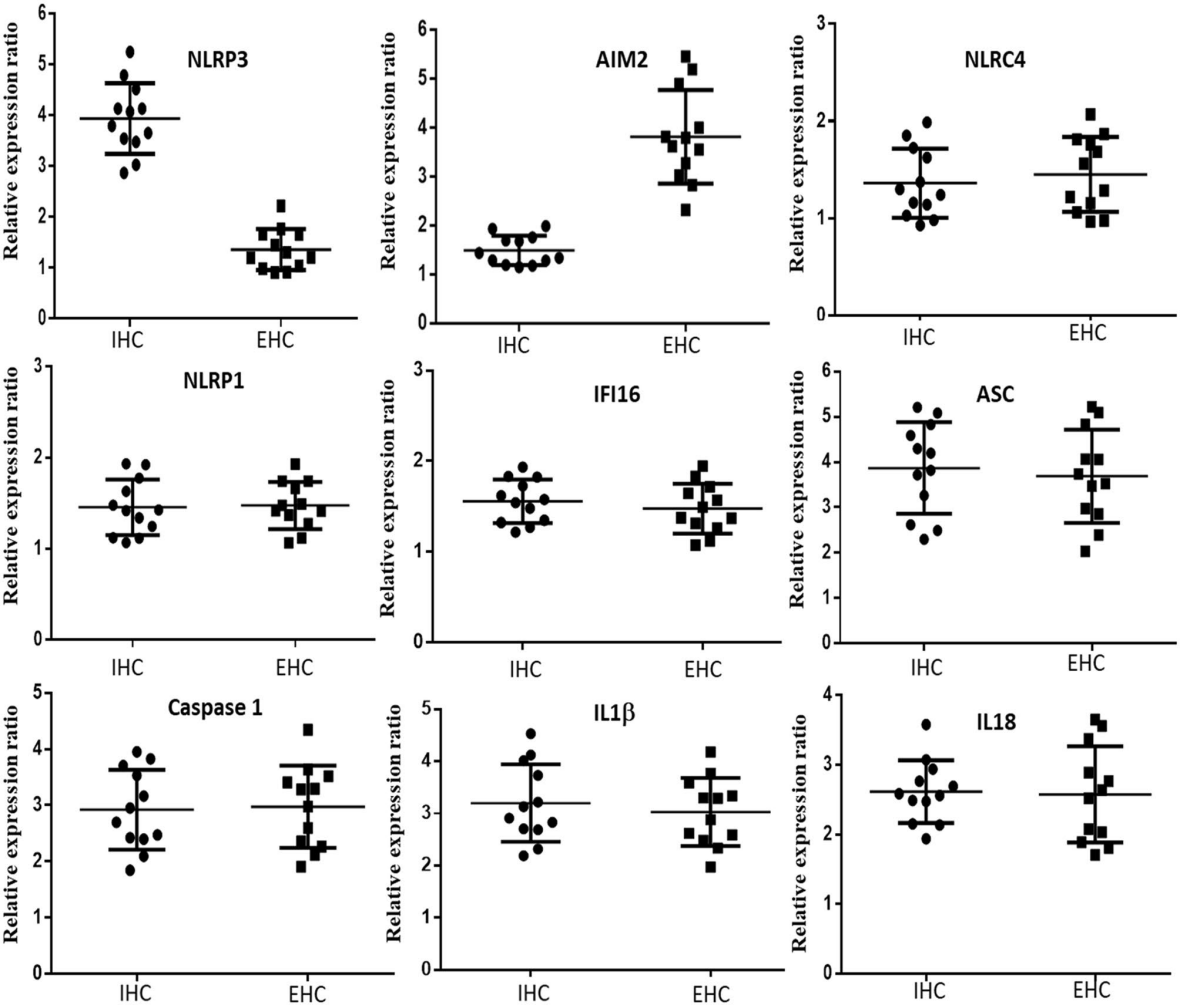
Differential mRNA expression of inflammasome pathway components in PBMCs from patients belonging to group 1 with either intrahepatic or extrahepatic cholestasis (N = 12). Their relative mRNA expression ratio with respect to group 4 (control group) in terms of fold change (2^−ΔΔCt^) were estimated by quantitative real time PCR. GAPDH mRNA served as internal control. (+ 1 was considered to be the baseline of control, with values greater than 1 suggesting positive fold change and values below 1 upto 0 as negative fold change).

Among the major activators of inflammasome pathway only NLRP3 showed high relative expression ratio in case of the IHC group and AIM2 in case of EHC group. For the other three activator components, NLRP1, NLRC4 and IFI16 there was no significant relative expression change in case of both IHC and EHC groups. The relative mRNA expression ratios of all the other components of the inflammasome pathway like ASC, Caspase1, IL1β and IL18 were significantly higher in case of both IHC and EHC groups in comparison to control. There were no significant differences in the expression of these four components among the IHC and EHC groups. Our findings confirmed that inflammasome was activated in case of patients with both intrahepatic and extrahepatic cholestasis but the initial activators were different. In case of intahepatic cholestasis the activation was through NLRP3 whereas in case of extrahepatic cholestasis the activation was through AIM2. A clear representation of our findings has been provided in Fig. 3. The detailed statistical analysis of the real time expression parameters has been provided in Supplementary Table 4.

### Phylogenetic analysis of HCMV *gN* and *gM* gene sequences from clinically isolated samples

The phylogenetic analysis utilizing 24 partial nucleotide sequences corresponding to each of HCMV *gM* and *gN* genes amplified from clinically isolated HCMV strains (12 from IHC patients and 12 from EHC patients) and 16 NCBI reference nucleotide sequences from different HCMV strains generated unique coalescent trees. The IHC and EHC groups clustered almostseparately in case of both the trees (*gN* based and *gM* based). The IHC groups in both cases were found to be more closely related to the reference strains but bearing distinct identity.

**Figure 4:**
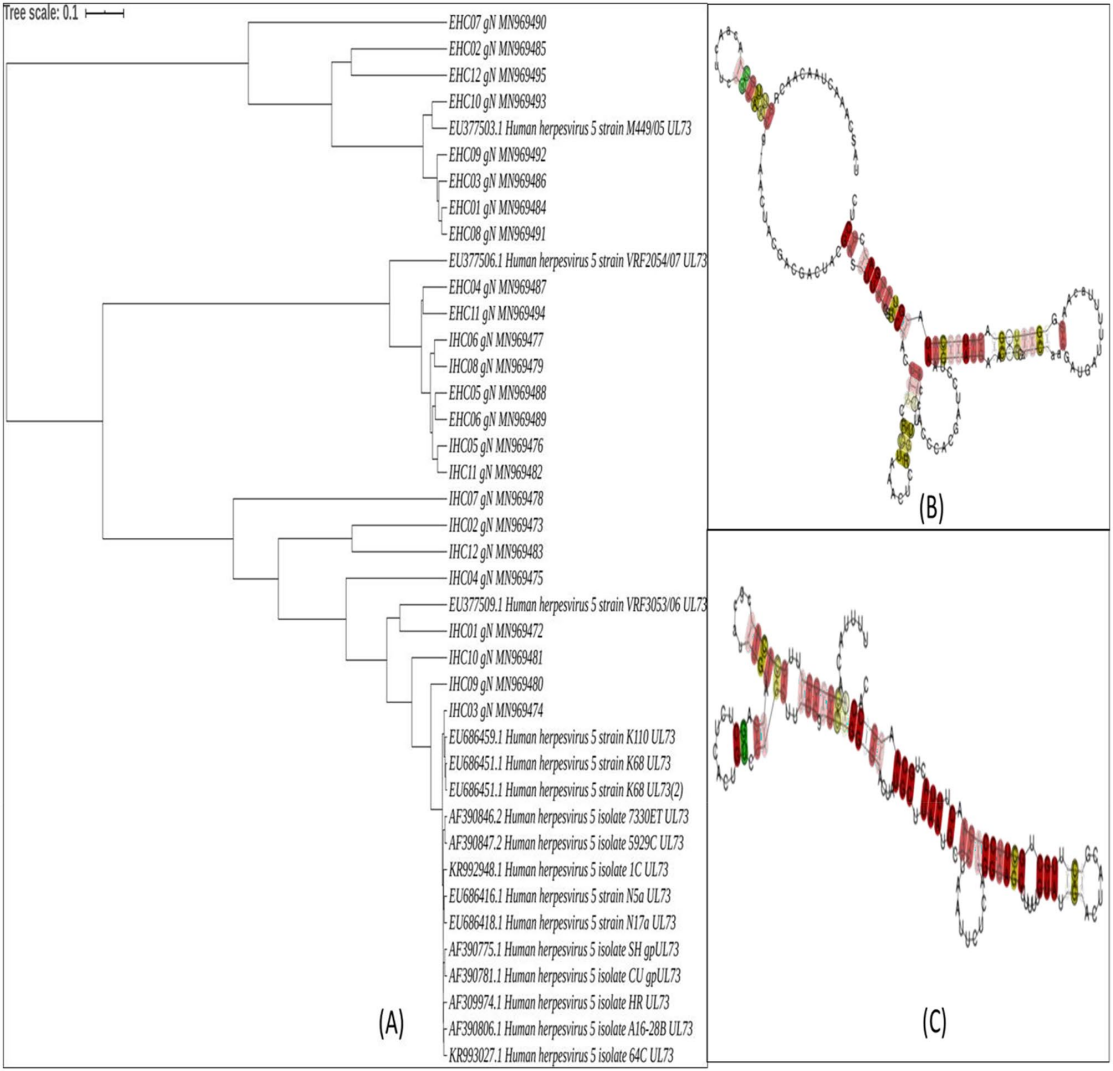
(**A**) Phylogenetic analysis of the partially sequenced HCMV gN gene from 24 clinical samples belonging to group 1 (12 IHC and 12 EHC samples) along with 16 standard reference gN gene sequences selected from NCBI database. (**B**) Structural classification of the conserved consensus gN mRNA local structure belonging to the clinical group with HCMV induced extrahepatic cholestasis (EHC). (**C**) Structural classification of the conserved consensus gN mRNA local structure belonging to the clinical group with HCMV induced intrahepatic cholestasis (IHC).

In case of the *gN* based tree, the IHC and EHC groups formed almost separate clusters, with the IHC cluster being more closely related to the reference strains. Eight of the 12 EHC clinical strains formed a totally separate cluster and showed nearest sequence similarity with only HHV5 reference strain M449. The other four EHC sequences showed close similarity with reference strain HHV5 VRF2054 and with four IHC strains. The rest of the eight IHC strains formed a completely separate cluster having close sequence similarity with all the other reference standard strains. The detailed analysis has been depicted in Fig. 4A.

**Figure 5:**
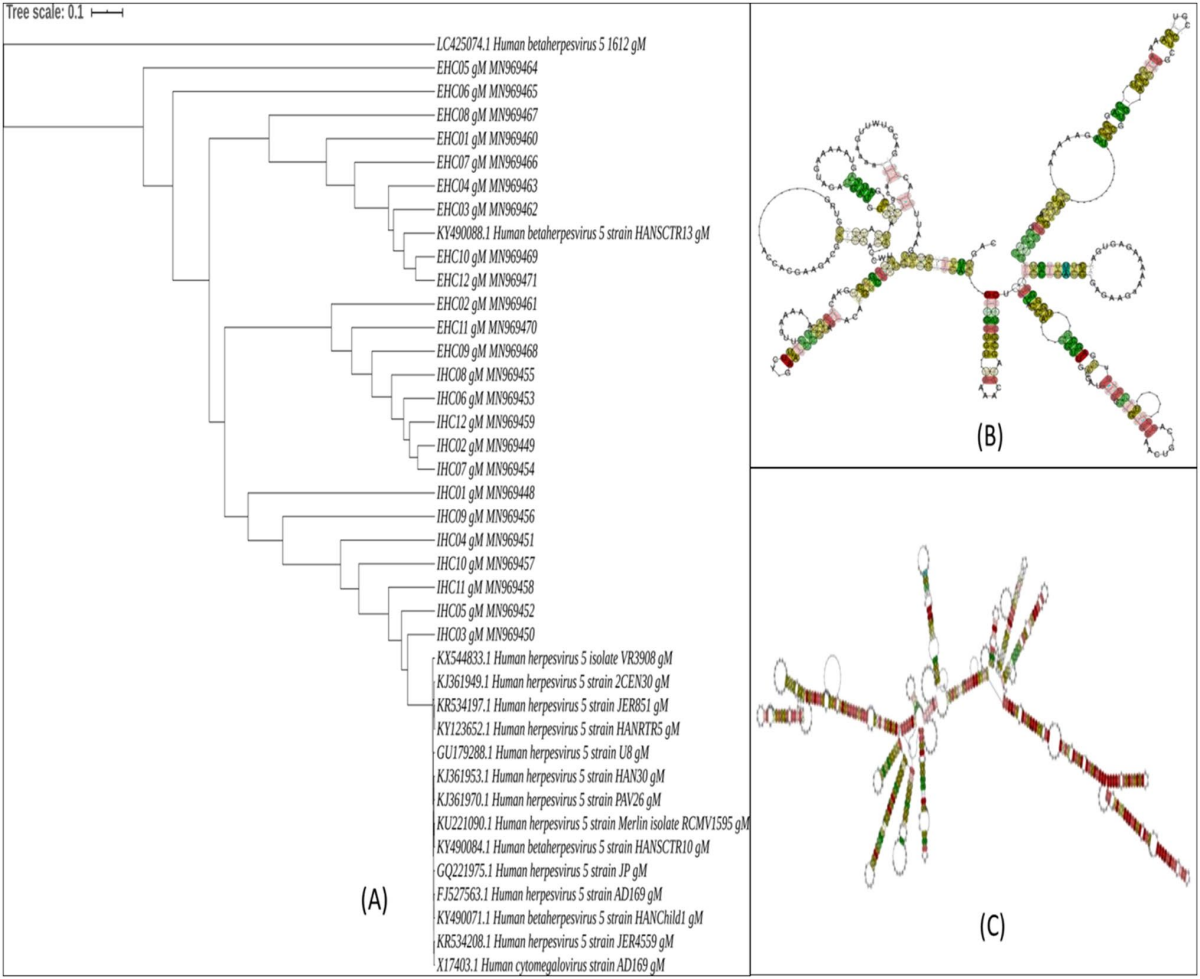
(**A**) Phylogenetic analysis of the partially sequenced HCMV gM gene from 24 clinical samples belonging to group 1 (12 IHC and 12 EHC samples) along with 16 standard reference gM gene sequences selected from NCBI database. (**B**) Structural classification of the conserved consensus gM mRNA local structure belonging to the clinical group with HCMV induced extrahepatic cholestasis (EHC). (**C**) Structural classification of the conserved consensus gM mRNA local structure belonging to the clinical group with HCMV induced intrahepatic cholestasis (IHC).

In case of the *gM* based tree, nine of the twelve EHC strains formed a separate cluster and showed similarity with two reference strains HHV5 1612 and HHV5 HANSCTR 13. The rest of the three EHC strains, 2, 9 and 11 formed a cluster with five IHC strains. The remaining seven IHC strains formed another separate cluster with the remaining reference strains. The detailed analysis has been depicted in Fig. 5A.

### Sequence and secondary structure based analyses to support the distinct phylogenetic pattern of the gene sequences for IHC and EHCgroups

To support our findings relevant to the distinctive phylogenetic patterns observed in case of clinical *gM* and *gN* gene sequences belonging to the IHC and EHC groups, we performed some analyses to ascertain their effective number of codons (ENc), codon adaptation index (CAI) and mRNA structure conservation.

The ENc values of clinical *gM* genes from IHC (Mean-51.593 ± 3.3) and EHC (Mean-55.919 ± 1.5) groups were much different and both were significantly higher than the ENc value of the gM genes from the reference HCMV strains (Mean = 45.528 ± 2.6). Our results indicated that there exists a significant difference in the codon usage bias among the two groups and the ENc value for the IHC group was more closely related to that of the reference strains. The influence of natural selection on the *gM* genes was inferred through CAI analysis. The mean ± SD values for CAI of clinical *gM* genes were 0.665 ± 0.022 and 0.692 ± 0.031 respectively for the IHC and EHC groups. The CAI of *gM* gene sequence belonging to the reference strains was 0.662 ± 0.019. The sequences of viral genes with higher CAIs are considered to be evolutionary more preferable over those with lower CAIs and hence more adaptable to their hosts. On the basis of CAI analysis of the *gM* genes from clinical IHC and EHC groups we observed that the values differed significantly among the two groups. The CAI value of the IHC group was more closely related to the reference CAI value indicating their close phylogenetic association.

When the same analyses were made with the *gN* gene sequences, the observed results were more or less similar as observed for *gM*. The ENc values of clinical *gN* genes from IHC (Mean-48.885 ± 2.7) and EHC (Mean-51.787 ± 1.2) groups were quite different and both were higher than the ENc value of the *gN* genes from the reference HCMV strains (Mean = 46.154 ± 1.2). The ENc value for the IHC group was found to be more closely related to that of the reference strains. Next in the case of *gN* too we made the CAI analysis, for IHC, EHC clinical groups and reference group. The mean ± SD values for CAI of clinical gN genes were 0.638 ± 0.033 and 0.654 ± 0.025 respectively for the IHC and EHC groups. The CAI of gN gene sequence belonging to the reference strains was 0.629 ± 0.011. In case of *gN* the CAI value of the IHC clinical group was also more closely related to the reference CAI value indicating their close phylogenetic association.

To understand if the mRNA structure of *gM* and *gN* genes play any role in the evolutionary dynamics of clinical HCMV strains and whether there exists any structural variation among the IHC and EHC clinical groups we have analyzed the conserved local structures between the two groups with respect to *gM* and *gN*. Although both the RNA consensus structures show high level of conservation but we were still able to decipher some significant structural variations in the *gM* and *gN* mRNAs between the two groups. The consensus *gN* mRNA structures for EHC and IHC groups has been depicted in Fig. 4B,C respectively while their corresponding structural alignments has been provided in Supplementary Figs. 2 and 3. The consensus *gM* mRNA structures for EHC and IHC groups has been depicted in Fig. 5B,C respectively while the corresponding structural alignments has been provided in Supplementary Figs. 4 and 5.

## Discussion

In this study we have shown that HCMV infection is associated with both intrahepatic and extrahepatic cholestasis in neonates and specified only four biomarkers (Cholesterol, GGT, ALP and TNFα) with exact cut-off values to distinguish between them thereby minimizing the labor, time constraint and cost of detection. These easily detectable blood markers will also help to track the status of progression and health condition of the neonates during follow-up. Further we have also identified that in patients with HCMV associated hepatic cholestasis, the inflammasome pathway is activated, causing a more aggravated liver damage. The extent of inflammasome activation was more or less same in case of both HCMV associated intrahepatic and extrahepatic cholestasis, but the initial activators were different; NLRP3 in case of IHC and AIM2 in case of EHC. Thus we were able to establish that HCMV infection can induce both intrahepatic and extrahepatic cholestasis among infants and there exists extensive physiological as well as immunological difference between the two types. A major question arose whether the same HCMV strains were infecting within the liver and outside of it or the strains infecting the two separate sites were genetically different? We sequenced and aligned partial regions of two very important HCMV glycoprotein genes (*UL100* or *gM* and *UL 73* or *gN*) isolated from 24 group 1 clinical samples (12 IHC and 12 EHC), along with 16 NCBI reference gene sequences for each gene. We performed two separate phylogenetic analyses for HCMV *gM* and *gN* gene sequences and found that in both cases the IHC and EHC groups formed almost separate phylogenetic clusters, with the IHC group more closely related to the majority of the reference strains. This may point towards the fact that a form of adaptive selection pressure is working on the clinical strains belonging to the two separate groups, causing selective mutations in their essential genes and diverging their phylogenetic lineage to some extent, thereby satisfying their needs for intra-host tissue tropism. We tried to explore whether the strains isolated from the two groups of patients (intrahepatic or extrahepatic) differed in their genetic lineages, and how much they vary compared to the NCBI reference strains. Our results from the phylogenetic analyses suggests that in a similar selection environment i.e. within or outside the liver, with a more or less constant selective pressure acting on them the strains are diverging similarly and hence the strains belonging to a particular group cluster together distinct from the strains belonging to the other group. To establish our findings we performed ENc and CAI analysis. The higher ENc values in clinical IHC/EHC samples for both *gM* and *gN*, compared to the reference strains indicate lower codon usage bias in the clinical viral genome. This might help the virus to survive within the competitive environment of its host as it will provide a significant adaptive fitness to the viral strains. ENc values for IHC samples were closer to that of the reference strains and are thus more closely related to them. Variation in the CAI value between the gM and gN genes of clinical and reference strains indicate that the gene of the clinical strains have different translational efficiency from that of the reference strains. We observed significant differences in the mean CAI value between the IHC and EHC groups which might indicate that the higher translational efficiency of these clinical strains might have categorically evolved to survive in specific host environments and under conditions of selective pressure inducing different types of disease manifestations. Selective site specific mutations in the *gM* and *gN* gene sequences can render functional variations in the interacting domains of the surface proteins thereby providing them with a slightly modified affinity for different surface markers at different tissue sites. Our assumptions were supported by the fact that we found significant structural variations in the mRNA secondary structures of both *gM/gN* when compared between the IHC and EHC specific clinical HCMV strains. There may be a significant chance for the variation in the mRNA secondary structure to be reciprocated as structural and functional changes in the respective proteins. These selective variations in the functional domain of the protein complex might play a fundamental role in the etiology of specific diseases in selective populations. These site specific variations if epitopic might also induce differential immune responses in a group (IHC or EHC) specific manner as validated from our comparative immunological profiling.

In conclusion our study suggests that the HCMV clinical strains infecting two different tissue sites (intrahepatic and extrahepatic) within the same host are genetically different, that have been selectively segregated in two separate phylogenetic clusters. These two separate groups have been able to somewhat modify the local physiological and immunological environment of the host as per their advantage and to suit their individual mode of infectivity.

## Methods

### Patient selection and data collection

In this study we have chosen neonates < 4 weeks old admitted to the Neonatal Intensive Care Unit of designated metropolitan hospitals. The main focus was on identifying neonates suffering from hepatic cholestasis with active Human Cytomegalovirus (HCMV) infection. This forms our main target population with 35 infants collected over a span of 2 years. Three other groups containing 25 infants in each were chosen as controls. The groups were divided in the following manner; Group 1 (Neonates with Hepatic cholestasis and active HCMV infection), Group 2 (Neonates without hepatic cholestasis but with active HCMV infection), Group 3 (Neonates with hepatic cholestasis but without HCMV infection) and Group 4 (Neonates without hepatic cholestasis and HCMV infection). Furthermore, the patients in group 1 were divided into three subgroups, 12 patients with intrahepatic cholestasis (IHC), 19 patients with extrahepatic cholestasis (EHC) and four patients with both intrahepatic and extrahepatic cholestasis (IHC + EHC). Data were collected at the time of admission, after 3 months and after 6 months from the date of admission at hospital. For detailed procedure follow Supplementary Appendix 1.

### Ethical considerations and patient’s consent

The present study and methodologies were approved by the scientific advisory committees (SAC) and certified by Institutional Ethics Committee (IEC) of ICMR NICED, Kolkata as well as all the respective hospitals in accordance with the 1964 Helsinki declaration. Written informed consents were taken after explaining all associated positive and negative aspects regarding the study to the parents of each participating patient in languages at least one of which they understand clearly. Our study included reports of all clinical examination, medical questionnaires, and personal family history of the patients as provided by the medical practitioner on duty. Confidentiality of the provided information was maintained properly as per the standard guidelines.

### Sample collection

EDTA (Ethylenediaminetetraacetic acid) anti coagulated peripheral blood (5–10 mL) was collected from patients in vacutainer tubes, processed immediately and serum was separated from the whole blood by centrifugation (1000×*g* for 10 min). Serum was quickly frozen at − 80 °C and stored until processed.

### DNA isolation from serum

DNA was isolated from the blood serum using QIamp DNA blood Mini Kit (Qiagen Inc., Hilden, Germany; 51106) as per manufacturer’sprotocol.

### HCMV qualitative PCR for virus detection

We designed the sequence of primers in the *UL 83* and *gB* regions of HCMV genome using primer 3 online software and using HCMV AD169 strain genome as reference. The primers were obtained from Eurofins Genomics India Pvt. Ltd. The primers were used to amplify the specific HCMV genes from the isolated clinical DNA samples. For detailed procedure follow Supplementary Appendix 2.

### Real-time PCR quantification of HCMV viral load

A quantitation standard curve was achieved by using six tenfold serial dilutions of a standard HCMV DNA with known viral load (copies/mL) purchased from ATCC. A conserved partial region of the HCMV *UL 75 (gH)* gene was amplified in from isolated viral DNA of patients in each case and Ct value was measured in a real time PCR instrument (ABI 7500-Applied Biosystems, USA). Viral load was determined by evaluating each unknown Ct value using the standard curve. For detailed procedure follow Supplementary Appendix 2.

### ELISA

Serum Tumor Necrosis Factor alpha (TNFα), Interferon gamma (IFN γ), Interlukin 6 (IL6), Interlukin 10 (IL10), Interlukin 1beta (IL1β), Transforming growth Factor beta (TGFβ), Interlukin 1 Receptor antagonist (IL1Ra), Interlukin 18 (IL18), Interlukin 8 (IL8) and C-reactive protein (CRP) levels in serum were measured by using enzyme-linked immunosorbent assay (ELISA) technique kits from Abcam Biotech Co., Cambridge, UK and G-Biosciences, Geno Technology Inc., USA. These assays detected only human cytokines and at very low serum concentrations. ELISA was performed as per manufacturer’s protocol.

### Isolation and culture of PBMCs

Peripheral blood mononuclear cells were isolated from the collected peripheral blood samples of each patient by Ficoll-Paque density gradient centrifugation using histopaque separation medium (Sigma-Aldrich; Merck KGaA). PBMCs from each group were cultured and proliferated separately with RPMI-1640 culture medium containing 10% FBS (both from Thermo Fisher Scientific, Inc.) for 24 h in vitro. Then, the PBMCs were harvested and used for RNA isolation. For detailed procedure follow Supplementary Appendix 2.

### RNA isolation and real-time polymerase chain reaction

Total RNA was extracted using TRIzol reagent (Invitrogen; Thermo Fisher Scientific, Inc.) from isolated PBMCs of patients according to the standardized protocol. Primescript 1st strand cDNA synthesis kit, TAKARA was used to convert RNA into cDNA following standard procedure. The cDNA was used for real-time polymerase chain reaction quantification. The real time expression ratios of mRNAs were determined using the 2^−ΔΔCt^ method for relative quantification. All real time primers were designed in the laboratory using primer-3 software and obtained from IDT technologies INC, India. The reaction parameters and conditions were standardized in the laboratory. All the primers that have been used in this study for real time expression have been listed in Supplementary Table 1. For detailed procedure follow Supplementary Appendix 2.

### Statistical analysis

Results were expressed as mean ± standard deviation, unless otherwise indicated. Differences between groups were compared by unpaired t-testing and one way analysis of variance (ANOVA) when distributions were normal. Non parametric tests were used if they were not normal. Bonferroni method was used with ANOVA for comparing between individual groups. Binary logistic regression was performed to assess statistical significance among two groups of interest. The level of significance (P value) was set at 5%. All P values were two tailed. All statistical analyses were carried out with SPSS software (version 16.0; SPSS, Inc., Chicago, IL, USA). Post-hoc sample size calculation analysis gave a power value of 74.3% with a total of 110 subjects and alpha value 0.05. All graphs were prepared using either SPSS 16.0 or graph pad prism version 6.0.

### Gene sequencing

Sequencing primers were designed to partially amplify a major conserved region of HCMV *UL 73 (gN)* and *UL100 (gM)* genes from 24 clinical samples belonging to group 1 (12 IHC and 12 EHC samples). Automated thermal cycler (Bioradinc.) was used to amplify the specified regions. These crude PCR products were outsourced to Agrigenome Labs Pvt. Ltd, India for Sanger sequencing. After obtaining the sequences they were analysed and submitted to NCBI gene data bank. These sequence data have been submitted to the GenBank databases under accession number MN969448–MN969471 for 24 *gM* nucleotide sequences and MN969472–MN969495 for 24 *gN* nucleotide sequences) available at https://www.ncbi.nlm.nih.gov/genbank. For further details follow Supplementary Appendix 2.

### Bioinformatics analysis

We had 24 partial nucleotide sequences corresponding to each of HCMV gM and gN genes amplified from clinically isolated HCMV strains and 16 NCBI reference nucleotide sequences from different HCMV strains available at https://www.ncbi.nlm.nih.gov/. The nucleotide sequences were aligned using MUSCLE^22^ and the poorly aligned regions with more than 20% gaps were trimmed using trimAl^23^. Coalescent trees were constructed through Bayesian analysis and the time of the most recent common ancestor (MRCA) for some strains and lineages was calculated using BEAST package V.2.6.0^24^ with Markov Chain Monte Carlo (MCMC) algorithm implemented in it. The XML file was generated in BEAUTi program using gamma parameter of site heterogeneity at 1,000,000 chain-lengths. Sampling prior and mean clock rate were estimated in Tracer software. iTOL^25^ was used to visualize the phylogenetic tree. The ENc (Effective number of codons)^26^ and CAI (Codon adaptation index)^27^ value for each sequence was calculated. The ENc value were calculated using the chips programmeavailable in EMBOSS package (https://www.bioinformatics.nl/cgi-bin/emboss/chips). An analysis of variance (ANOVA) test was performed to determine whether the ENc values were significantly different between EHC and IHC. CAI programmer available in EMBOSS package (https://www.bioinformatics.nl/cgi-bin/emboss/cai) was used to calculate the CAI. LocARNA was used to generate the local structural alignment and consensus mRNA secondary structures^28,29^. The highly conserved residues in the consensus structure were highlighted in red. RNAscClust was used for performing structural classification of mRNA between two groups^30^. The detailed analysis and procedure has been described in Supplementary Appendix 3.

## Supplementary information

Supplementary Information.

## Abbreviations

HCMV: Human Cytomegalovirus
HC: Hepatic cholestasis
SGOT: Serum glutamic oxaloacetic transaminase
SGPT: Serum glutamic pyruvic transaminase
ALP: Alkaline phosphatase
GGT: Gamma glutamyltransferase
TGL: Triglycerides
LDL: Low density lipoprotein
HDL: High density lipoprotein
TNFα: Tumor necrosis factor alpha
IFNγ: Interferon gamma
IL6: Interlukin 6
IL10: Interlukin 10
IL1β: Interlukin 1 beta
TGFβ: Transforming growth factor beta
IL1Ra: Interlukin 1 receptor antagonist
IL18: Interlukin 18
IL8: Interlukin 8
CRP: C-Reactive protein
MCP1: Monocyte chemoattractant protein 1
MIP1α: Macrophage inflammatory protein 1-alpha
IL2: Interlukin 2
RIG1: Retinoic acid-inducible gene 1
RANTES: Regulated on Activation Normal T Cell Expressed and Secreted (RANTES)
NLRP1: NLR family pyrin domain containing 1
NLRP3: NLR family pyrin domain containing 3
NLRC4: NLR family CARD domain-containing protein 4
AIM2: Absent in melanoma 2
IFI16: Interferon gamma inducible protein 16
ASC: Apoptosis-associated speck-like protein containing a CARD domain
ROC: Receiver-operating characteristic
AUC: Area under the curve
ANOVA: Analysis of variance

## References

[1] Feldman, A. G. & Sokol, R. J. Neonatal cholestasis. *Neoreviews* **14**, 10.1542/neo.14-2-e63. (2013). 10.1542/neo.14-2-e63 PMC3827866 24244109

[2] Götze T, Blessing H, Grillhösl C, Gerner P, Hoerning A (2015). Neonatal cholestasis—Differential diagnoses, current diagnostic procedures, and treatment. Front. Pediatr..

[3] Poley, J. Syndromes of neonatal cholestasis. in Gracey M, Burke V, eds. *Pediatr. Gastroenterol. Hepatol. 3. ed*. 566–93 (Bost. Blackwell Sci., 1993).

[4] Suchy FJ (2004). Neonatal cholestasis. Pediatr. Rev..

[5] Fischler B, Ehrnst A, Forsgren M, Orvell C, Nemeth A (1998). The viral association of neonatal cholestasis in Sweden: A possible link between cytomegalovirus infection and extrahepatic biliary atresia. J. Pediatr. Gastroenterol. Nutr..

[6] Chang MH (1992). Polymerase chain reaction to detect human cytomegalovirus in livers of infants with neonatal hepatitis. Gastroenterology.

[7] Lurie M, Elmalach I, Schuger L, Weintraub Z (1987). Liver findings in infantile cytomegalovirus infection: Similarity to extrahepatic biliary obstruction. Histopathology.

[8] Ozkan TB, Mistik R, Dikici B, Nazlioglu HO (2007). Antiviral therapy in neonatal cholestatic cytomegalovirus hepatitis. BMC Gastroenterol..

[9] Aaradhana S, Ravi S (2018). Congenital cytomegalovirus infection presenting as severe conjugated hyperbilirubinemia on first day of life. Int. J. Pediatr. Res..

[10] Martinon F, Burns K, Tschopp J (2002). The inflammasome: A molecular platform triggering activation of inflammatory caspases and processing of proIL-beta. Mol. Cell.

[11] Schroder K, Tschopp J (2010). The Inflammasomes. Cell.

[12] de Zoete MR, Palm NW, Zhu S, Flavell RA (2014). Inflammasomes. Cold Spring Harb. Perspect. Biol..

[13] Strowig T, Henao-Mejia J, Elinav E, Flavell R (2012). Inflammasomes in health and disease. Nature.

[14] Sutterwala FS, Haasken S, Cassel SL (2014). Mechanism of NLRP3inflammasome activation. Ann. N. Y. Acad. Sci..

[15] González-Navajas JM, Lee J, David M, Raz E (2012). Immunomodulatory functions of type I interferons. Nat. Rev. Immunol..

[16] Wree A (2014). NLRP3inflammasome activation results in hepatocyte pyroptosis, liver inflammation, and fibrosis in mice. Hepatology.

[17] Huang Y (2017). Human cytomegalovirus triggers the assembly of AIM2inflammasome in THP-1-derived macrophages. J. Med. Virol..

[18] Mach M, Kropff B, Dal Monte P, Britt W (2000). Complex formation by human cytomegalovirus glycoproteins M (gpUL100) and N (gpUL73). J. Virol..

[19] Kari B, Gehrz R (1992). A human cytomegalovirus glycoprotein complex designated gC-II is a major heparin-binding component of the envelope. J. Virol..

[20] Kropff B (2012). Glycoprotein N of human cytomegalovirus protects the virus from neutralizing antibodies. PLOSPathog..

[21] Kari B, Goertz R, Gehrz R (1990). Characterization of cytomegalovirus glycoproteins in a family of complexes designated gC-II with murine monoclonal antibodies. Arch. Virol..

[22] Edgar RC (2004). MUSCLE: Multiple sequence alignment with high accuracy and high throughput. Nucleic Acids Res..

[23] Capella-Gutiérrez S, Silla-Martínez JM, Gabaldón T (2009). trimAl: A tool for automated alignment trimming in large-scale phylogenetic analyses. Bioinformatics.

[24] Bouckaert R (2019). BEAST 2.5: An advanced software platform for Bayesian evolutionary analysis. PLOS Comput. Biol..

[25] Letunic I, Bork P (2019). Interactive Tree Of Life (iTOL) v4: Recent updates and new developments. Nucleic Acids Res..

[26] Fuglsang A (2004). The ‘effective number of codons’ revisited. Biochem. Biophys. Res. Commun..

[27] Sharp PM, Li W-H (1987). The codon adaptation index-a measure of directional synonymous codon usage bias, and its potential applications. Nucleic Acids Res..

[28] Kiening M (2019). Conserved secondary structures in viral mRNAs. Viruses.

[29] Will S, Joshi T, Hofacker IL, Stadler PF, Backofen R (2012). LocARNA-P: Accurate boundary prediction and improved detection of structural RNAs. RNA.

[30] Miladi M (2017). RNAscClust: Clustering RNA sequences using structure conservation and graph based motifs. Bioinformatics.

