# Supplementary material for: Insight into the distinctive paradigm of Human Cytomegalovirus associated intrahepatic and extrahepatic cholestasis in neonates
Source: Sci Rep. 2020 Sep 28;10:15861. doi: 10.1038/s41598-020-73009-z (PMC7522230; doi:10.1038/s41598-020-73009-z)
Supplement: Supplementary file 1 — Supplementary Information. [file 41598_2020_73009_MOESM1_ESM.docx]

**Supplementary data**

**Title:** Insight into the distinctive paradigm of Human Cytomegalovirus associated intrahepatic and extrahepatic cholestasis in neonates

Aroni Chatterjee^1^, Sumit Mukherjee^2#^, Biswanath Basu^3#^, Debsopan Roy^1^, Rivu Basu^4^, Hiya Ghosh^5^, Lopamudra Mishra^6^, Mala Bhattacharya^6^, Nilanjan Chakraborty^1@^

# Equal contribution

1. Virus Research Laboratory, ICMR-NICED, Kolkata-700010, India
2. Azrieli Faculty of Medicine, Bar-Ilan University, Safed, Israel
3. Department of Paediatrics, N.R.S. Medical College & Hospital, Kolkata-700014, India.
4. Department of Community Medicine, R.G.Kar Medical College and Hospital, Kolkata-700004, India
5. Department of Endocrinology and Metabolism, IPGMER & SSKM Hospital, Kolkata-700020, India
6. Department of Paediatrics, Dr. B.C.Roy Post graduate institute of paediatric Sciences,

Kolkata-700054, India

**@Correspondence:** Dr. Nilanjan Chakraborty, Scientist F, ICMR-National Institute of Cholera and Enteric Diseases, Kolkata, GB4, ID & BG hospital campus, Dr. S.C Banerjee road, Beliaghata, Kolkata-700010, West Bengal, India. Phone: +919163785518. Email: [nilanjan_19@yahoo.com](mailto:nilanjan_19@yahoo.com).

**Supplementary tables**

**Supplementary table 1: The detailed list of primers used for quantitative real time PCR**

| **Primer Name** | **Forward Primer** | **Reverse Primer** |
| --- | --- | --- |
| **MCP1** | GAAAGTCTCTGCCGCCCTT | GGTGACTGGGGCATTGATTG |
| **MIP1α** | GCTCTCTGCAACCAGTTCTCT | GGCTTCGCTTGGTTAGGAAGA |
| **RANTES** | AGCAGTCGTCCACAGGTCAA | ACACTTGGCGGTTCTTTCGG |
| **TGFβ** | ATTGAGGGCTTTCGCCTTAG | TAGTGAACCCGTTGATGTCC |
| **TNF α** | GCTGCACTTTGGAGTGATCG | GTCACTCGGGGTTCGAGAAG |
| **IFN γ** | TTCCTTGATGGTCTCCACAC | GGTCATTCAGATGTAGCGGA |
| **IL2** | ACAGGATGCAACTCCTGTCT | TGCTCCAGTTGTAGCTGTGT |
| **RIG 1** | CCTACCTACATCCTGAGCTACAT | TCTAGGGCATCCAAAAAGCCA |
| **NLRP3** | GAATGCCTTGGGAGACTCAG | GAGTACCGAGGACAAAGCTG |
| **Caspase 1** | CTTCCCGAATACCATGAGAC | AAGAACTGGAGCTGAGGTTG |
| **IL1 β** | GAGCACTTCATCTGTTTAGGGC | AGCTCGCCAGTGAAATGATGG |
| **ASC** | CCTTGGACCTCACCGACAA | ATGTCGCGCAGCACGTTAG |
| **AIM 2** | TAGCGCCTCACGTGTGTTAG | TTGAAGCGTGTTGATCTTCG |
| **NLRC4** | TTCGTCTTCTTCCTCCGTCT | ATGTCTGCTTCCTGATTGTG |
| **NLRP 1** | ACTCTCCCTCATTCCCCTAC | GCTGTCTCAAAACCCT TCTC |
| **IL 18** | ATGGCTGCTGAACCAGTAGAAG | CAGCCATACCTCTAGGCTGGC |
| **IFI 16** | GAAGTGCCAGCGTAACTCCTAA | TGATTGTGGTCAGTCGTCCAT |

**Supplementary table 2:** A comparative analysis of all the non-significant demographic and serum biochemical factors differentiating group 1 from group 2 and group 3. Mean+SD values were calculated and one way ANOVA was performed using Bonferroni method (Post-hoc analysis) for comparison with respect to Group 1.

| **Parameters** | | **Mean+/-SD** | **Significance**  **(P value)** | **95% Confidence Interval**  **Lower Upper** | |
| --- | --- | --- | --- | --- | --- |
| **Age (Days)** | **Group 1** | **21.31+4.75** | **Constant** | | |
|  | **Group 2** | **22.6+4.61** | **1.000** | **-5.48** | **2.91** |
|  | **Group 3** | **21.87+5.59** | **1.000** | **-4.75** | **3.64** |
|  | **Group 4** | **21.15+5.34** | **1.000** | **-3.65** | **3.97** |
| **Weight (Gms)** | **Group 1** | **2341.8**+**345.1** | **Constant** | | |
|  | **Group 2** | **2312.8+220.5** | **1.000** | **-239.62** | **297.49** |
|  | **Group 3** | **2456+307.5** | **1.000** | **-382.75** | **154.35** |
|  | **Group 4** | **2549+350** | **0.145** | **-451.18** | **36.68** |
| **Gestational age at delivery (Weeks)** | **Group 1** | **36.03+1.85** | **Constant** | | |
|  | **Group 2** | **35.9+1.5** | **1.000** | **-1.37** | **1.56** |
|  | **Group 3** | **36.4+1.55** | **1.000** | **-1.9** | **1.03** |
|  | **Group 4** | **36.1+1.86** | **1.000** | **-1.4** | **1.26** |
| **Age of Mother (Years)** | **Group 1** | **24.4+1.29** | **Constant** | | |
|  | **Group 2** | **22.9+3.1** | **1.000** | **-1.72** | **4.71** |
|  | **Group 3** | **24.07+3.43** | **1.000** | **-2.85** | **3.58** |
|  | **Group 4** | **23.6+3.8** | **1.000** | **-2.14** | **3.70** |
| **Chest Circumference (Cms)** | **Group 1** | **32.1+/-1.76** | **Constant** | | |
|  | **Group 2** | **32.6+/-1.29** | **1.000** | **-1.909** | **0.938** |
|  | **Group 3** | **33.3+/-1.37** | **0.138** | **-2.642** | **0.204** |
|  | **Group 4** | **34.35+/-1.73** | **0.000** | **-3.528** | **0.943** |
| **Length (Cms)** | **Group 1** | **45.8+1.79** | **Constant** | | |
|  | **Group 2** | **45.1+1.86** | **0.058** | **0.123** | **3.382** |
|  | **Group 3** | **46.3+2.28** | **1.000** | **-2.077** | **1.182** |
|  | **Group 4** | **46.8+2** | **0.540** | **-2.419** | **0.541** |
| **Bicarbonate (mEq/dl)** | **Group 1** | **27.5+5.54** | **Constant** | | |
|  | **Group 2** | **23.5+4.52** | **0.062** | **-0.1160** | **8.1488** |
|  | **Group 3** | **30.7+4.63** | **0.235** | **-7.3360** | **0.9288** |
|  | **Group 4** | **25.6+4.3** | **1.000** | **-1.8237** | **5.683** |
| **Ammonia (μmol/L)** | **Group 1** | **67.5+5.11** | **Constant** | | |
|  | **Group 2** | **66+6.98** | **1.000** | **-4.1005** | **7.2159** |
|  | **Group 3** | **49.4+4.52** | **1.000** | **12.492** | **23.809** |
|  | **Group 4** | **63.09+9.9** | **1.000** | **-0.6721** | **9.606** |
| **Hematocrit (%)** | **Group 1** | **45.58+8.43** | **Constant** | | |
|  | **Group 2** | **45.19+4.28** | **1.000** | **-5.445** | **6.223** |
|  | **Group 3** | **47.8+7.31** | **1.000** | **-8.1302** | **3.539** |
|  | **Group 4** | **46.26+5.28** | **1.000** | **-5.979** | **4.619** |
| **Chloride** | **Group 1** | **108.6+10.3** | **Constant** | | |

| **(mmol/L)** | **Group 2** | **112.4+6.89** | **1.000** | **-11.769** | **4.2746** |
| --- | --- | --- | --- | --- | --- |
|  | **Group 3** | **108.9+6.7** | **1.000** | **-8.3627** | **7.681** |
|  | **Group 4** | **107.8+11.6** | **1.000** | **-6.542** | **8.029** |
| **Urea (mmol/L)** | **Group 1** | **6.2+0.72** | **Constant** | | |
|  | **Group 2** | **5.9+1.05** | **0.991** | **-0.4192** | **0.9967** |
|  | **Group 3** | **6.02+0.92** | **0.962** | **-0.5192** | **0.8967** |
|  | **Group 4** | **6.04+0.81** | **1.000** | **-0.4811** | **0.8049** |
| **Creatinine (mg/dl)** | **Group 1** | **2.64+0.56** | **Constant** | | |
|  | **Group 2** | **2.4+0.4** | **1.000** | **-0.2095** | **0.6084** |
|  | **Group 3** | **2.38+0.49** | **0.535** | **-0.1489** | **0.6690** |
|  | **Group 4** | **2.23+0.38** | **0.024** | **0.0355** | **0.7784** |
| **Glucose (mmol/L)** | **Group 1** | **6.36+1.44** | **Constant** | | |
|  | **Group 2** | **6.27+0.97** | **1.000** | **-0.8549** | **1.021** |
|  | **Group 3** | **5.68+0.66** | **0.323** | **-0.2596** | **1.616** |
|  | **Group 4** | **5.98+0.79** | **1.000** | **-0.4801** | **1.224** |

**Supplementary table 3:** A comparative analysis of mean relative expression ratio (2^-ΔΔCt^) of different immunological markers quantified using real time PCR. Mean+SD values were calculated and one way ANOVA was performed to estimate the significance among group1 (N=10), group 2 (N=10) and group 3 (N=10). Group 4 was taken as the control.

| **Markers** | **Mean+SD** | **P value** | **R2** |
| --- | --- | --- | --- |
| NLRP3 | Group 1- 2.301+0.62 | <0.001 | 0.704 |
|  | Group 2- 1.2+0.348 |  |  |
|  | Group 3- 1.8+0.224 |  |  |
| Caspase1 | Group 1- 3.24+0.751 | < 0.001 | 0.783 |
|  | Group 2- 1.44+0.337 |  |  |
|  | Group 3- 1.48+0.522 |  |  |
| ASC | Group 1- 2.691+0.75 | < 0.001 | 0.815 |
|  | Group 2- 1.332+0.34 |  |  |
|  | Group 3- 1.265+0.31 |  |  |
| IL1β | Group 1- 3.32+0.91 | < 0.001 | 0.816 |
|  | Group 2- 1.28+0.31 |  |  |
|  | Group 3- 1.27+0.43 |  |  |
| MCP1 | Group 1- 2.45+0.78 | < 0.001 | 0.538 |
|  | Group 2- 2.41+0.97 |  |  |
|  | Group 3- 1.32+0.36 |  |  |
| MIP1α | Group 1- 2.49+1.12 | 0.8 | 0.016 |
|  | Group 2- 2.28+0.65 |  |  |
|  | Group 3- 2.32+0.46 |  |  |
| RANTES | Group 1- 1.27+0.46 | 0.28 | 0.089 |
|  | Group 2- 1.45+0.37 |  |  |
|  | Group 3- 1.35+0.28 |  |  |
| IFNγ | Group 1- 3.06+0.71 | <0.001 | 0.706 |
|  | Group 2- 3.1+0.89 |  |  |
|  | Group 3- 1.28+0.39 |  |  |
| TGFβ | Group 1- 1.32+0.44 | 0.389 | 0.674 |
|  | Group 2- 1.25+0.65 |  |  |
|  | Group 3- 1.37+0.41 |  |  |
| TNFα | Group 1- 4.22+1.15 | 0.004 | 0.34 |
|  | Group 2- 2.96+1.05 |  |  |
|  | Group 3- 2.75+0.88 |  |  |
| RIG1 | Group 1- 1.21+0.2 | 0.772 | 0.018 |
|  | Group 2- 1.17+0.25 |  |  |
|  | Group 3- 1.24+0.36 |  |  |
| IL2 | Group 1- 0.83+0.19 | <0.001 | 0.545 |
|  | Group 2- 0.84+0.28 |  |  |
|  | Group 3- 1.24+0.31 |  |  |

**Supplementary table 4:** A comparative analysis of mean relative expression ratio (2^-ΔΔCt^) of different inflammasome pathway markers quantified using real time PCR. Mean+SD values were calculated and student’s t test was performed to estimate the significance among group 1 patients with either IHC (N=12) or EHC (N=12). Group 4 was taken as the control.

| **Markers** | **Mean+SD** | **P value** | **95% CI** |
| --- | --- | --- | --- |
| NLRP3 | IHC- 3.39+0.694 | <0.001 | -3.067 to -2.091 |
|  | EHC- 1.353+0.4 |  |  |
| Caspase1 | IHC- 2.921+0.81 | 0.85 | -0.0557 to 0.664 |
|  | EHC- 2.975+0.75 |  |  |
| ASC | IHC- 3.86+1.02 | 0.67 | -1.041 to 0.684 |
|  | EHC-3.69+1.03 |  |  |
| IL1β | IHC- 3.2+0.78 | 0.56 | -0.765 to 0.423 |
|  | EHC- 3.02+0.71 |  |  |
| AIM2 | IHC- 1.497+0.3 | <0.001 | -1.698 to 2.945 |
|  | EHC-3.879+0.96 |  |  |
| IFI16 | IHC- 1.56+0.34 | 0.45 | -0.297 to 0.136 |
|  | EHC- 1.47+0.37 |  |  |
| NLRP1 | IHC- 1.457+0.303 | 0.87 | -0.219 to 0.258 |
|  | EHC- 1.476+0.26 |  |  |
| NLRC4 | IHC- 1.361+0.354 | 0.557 | -0.223 to 0.402 |
|  | EHC-1.451+0.384 |  |  |
| IL18 | IHC- 2.61+0.55 | 0.87 | -0.537 to 0.458 |
|  | EHC- 2.57+0.69 |  |  |

**Supplementary figures**


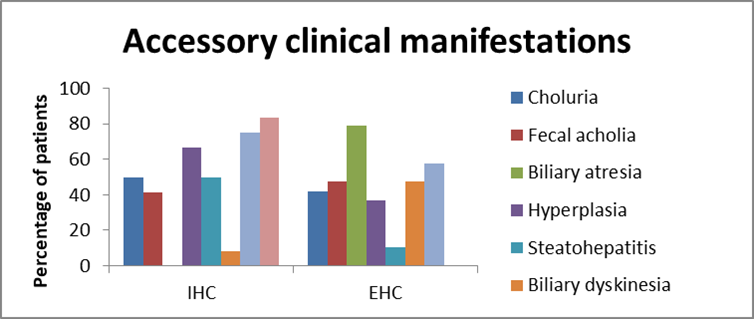


**Supplementary figure 1: A comparative assessment of accessory clinical conditions present among the infants with HCMV associated intrahepatic and extrahepatic cholestasis.**

**
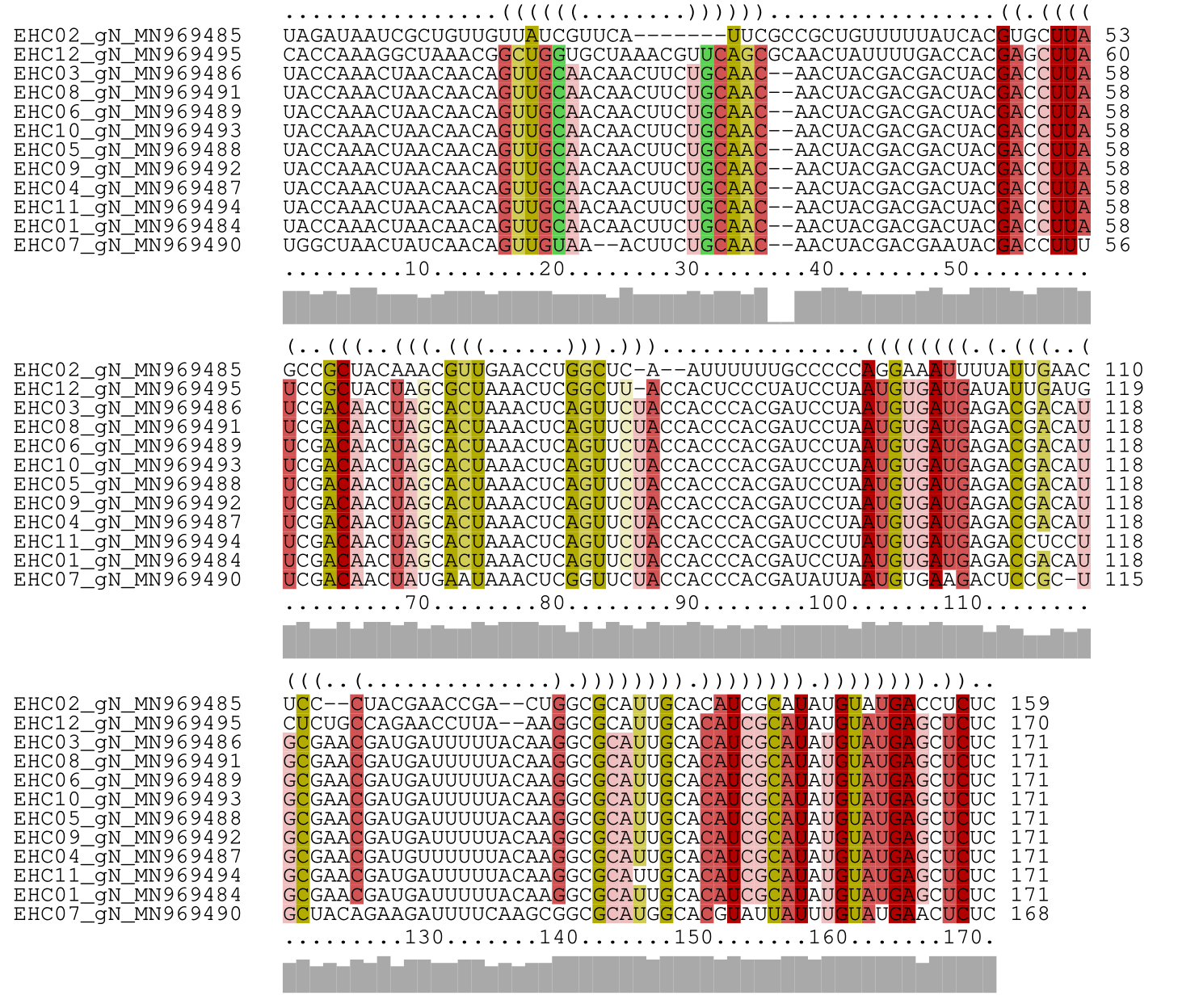
**

**Supplementary figure 2: A detailed depiction of the structural alignment for gN gene among the HCMV strains belonging to the EHC group.**


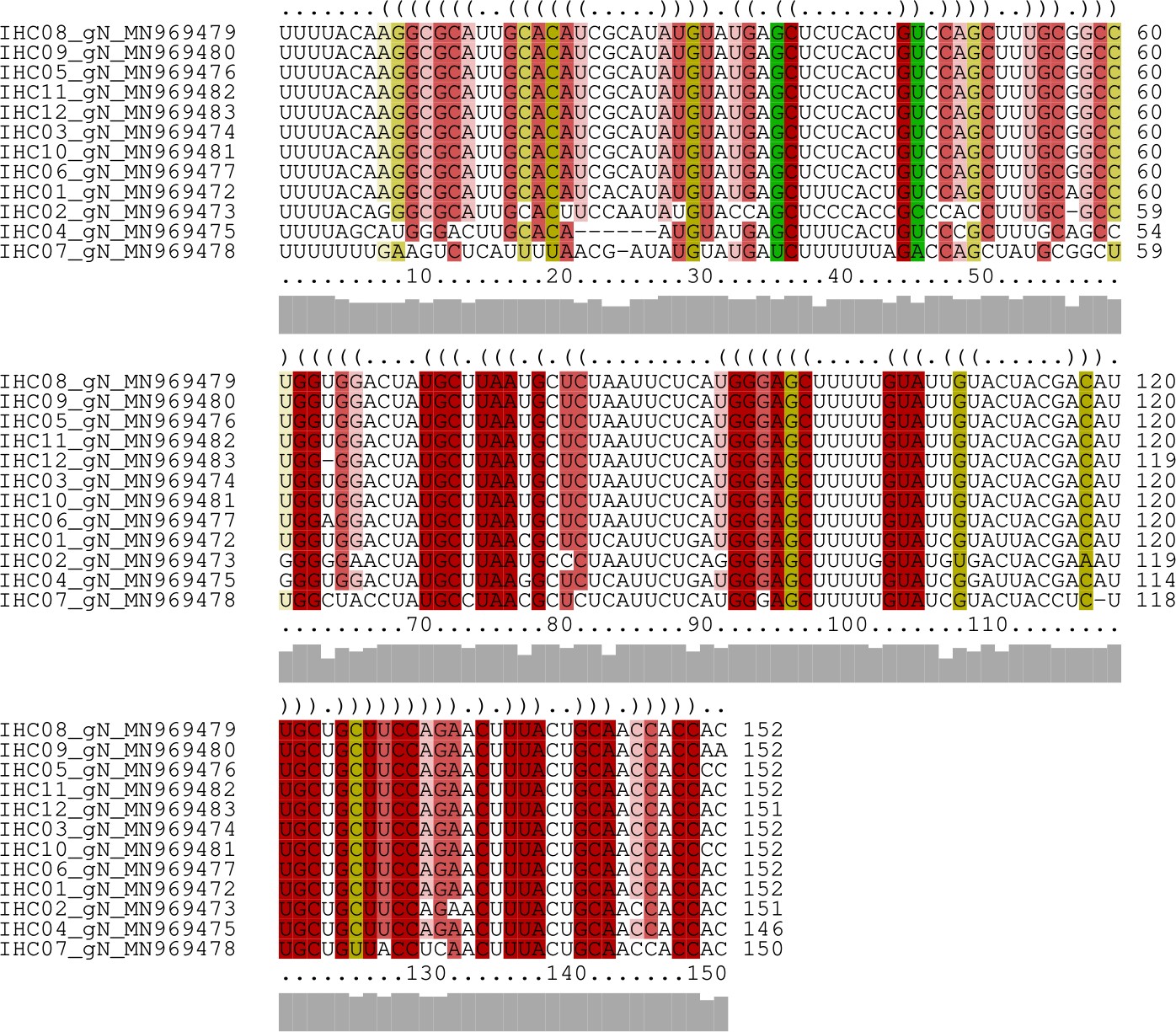


**Supplementary figure 3: A detailed depiction of the structural alignment for gN gene among the HCMV strains belonging to the IHC group.**


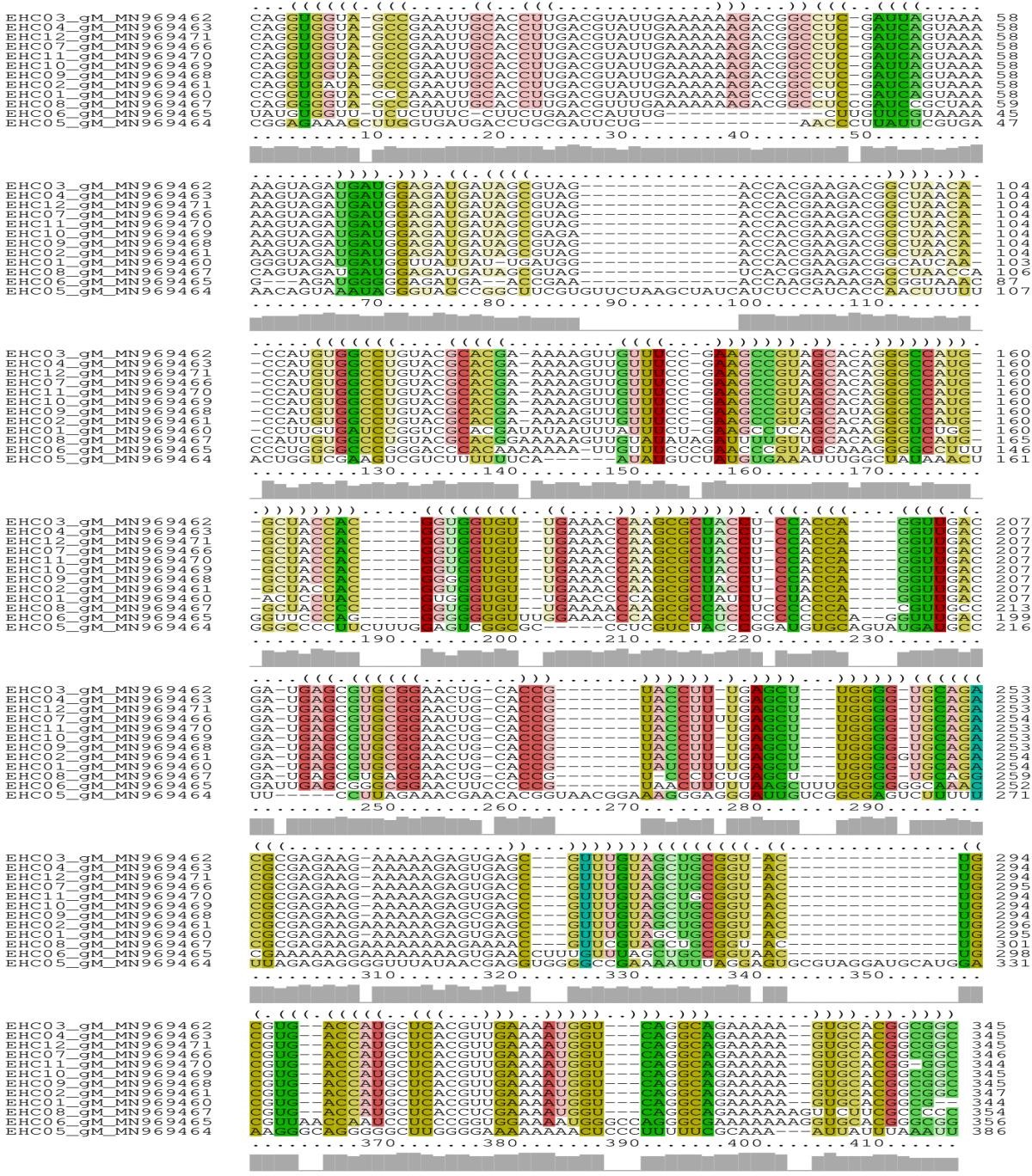


**Supplementary figure 4: A detailed depiction of the structural alignment for gM gene among the HCMV strains belonging to the EHC group.**


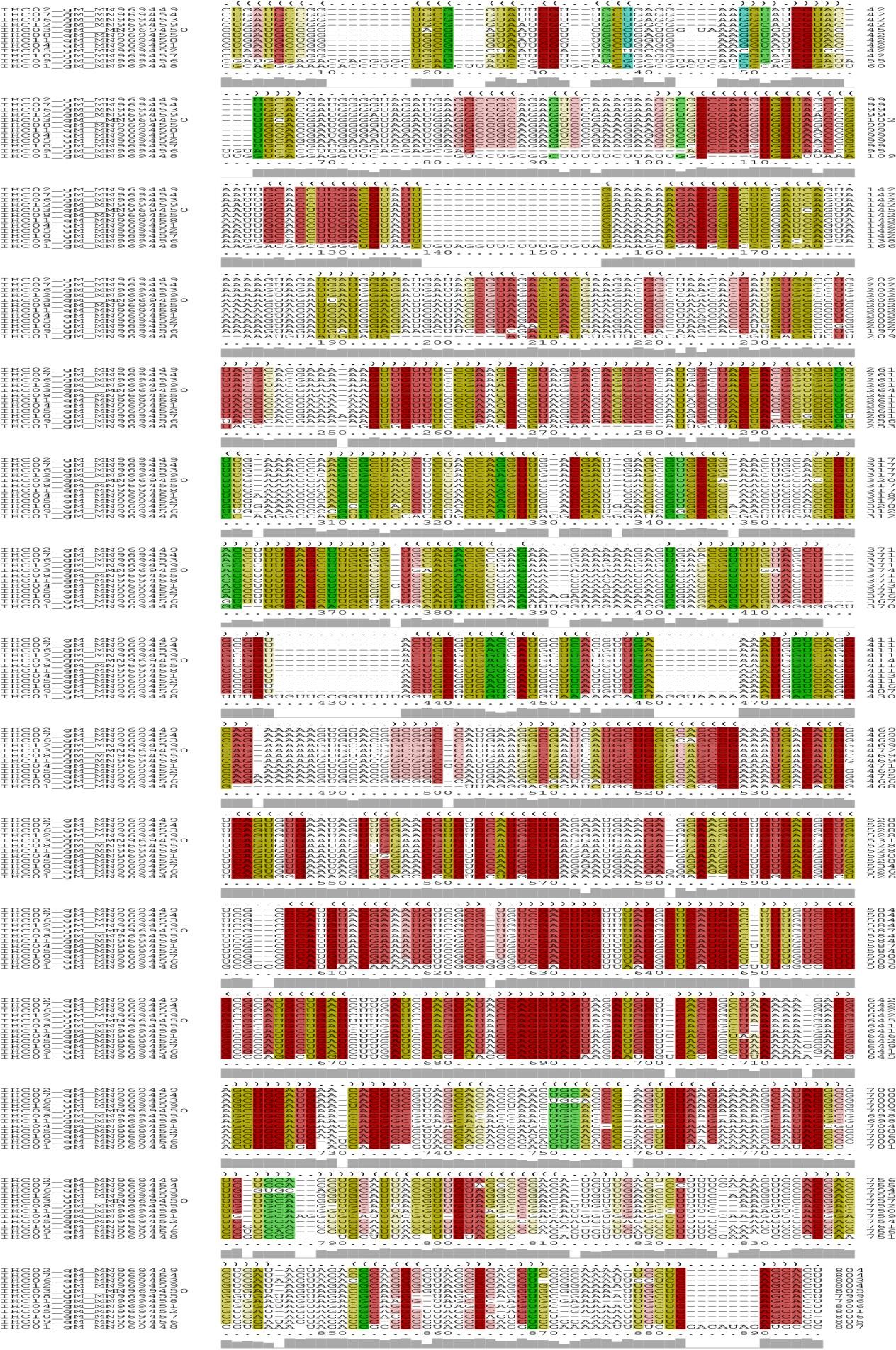


**Supplementary figure 5: A detailed depiction of the structural alignment for gM gene among the HCMV strains belonging to the IHC group.**

Supplementary Appendix 1 Patient Selection

In this study we have chosen neonates < 4 weeks old admitted to the Neonatal Intensive Care Unit of designated metropolitan hospitals. The main focus was on identifying neonates suffering from hepatic cholestasis with active Human Cytomegalovirus (HCMV) infection. This forms our main target population with 35 infants collected over a span of two years. Three other groups containing 25 infants in each were chosen as controls. The groups were divided in the following manner; Group 1 (Neonates with Hepatic cholestasis and active HCMV infection), Group 2 (Neonates without hepatic cholestasis but with active HCMV infection), Group 3 (Neonates with hepatic cholestasis but without HCMV infection) and Group 4 (Neonates without hepatic cholestasis and HCMV infection). Furthermore, the patients in group 1 were divided into three subgroups, 12 patients with intrahepatic cholestasis (IHC), 19 patients with extrahepatic cholestasis (EHC) and 4 patients with both intrahepatic and extrahepatic cholestasis (IHC+EHC). A flow chart of patient selection has been provided in figure 1.

Inclusion Criteria

All patients were chosen by the medical practitioners after carefully monitoring their health conditions, medical reports and clinical profile. Neonates with hepatic cholestasis were diagnosed having conjugated hyperbillirubinemia and high levels of ALP, SGOT and SGPT in their blood serum. Utrasound imaging, hepatobiliary scan and scintigraphic techniques as well as liver biopsy in some cases were performed to confirm hepatic cholestasis along with differentiating between intrahepatic and extrahepatic causes. HCMV positive patients were identified by amplifying HCMV positive DNA in blood by PCR. Molecular diagnosis of rubella, toxoplasma, HSV, HBV, HCV, HAV, bacterial infections etc. were performed and only patients with negative results for all but positive for HCMV were selected.

Exclusion criteria

Moribund patients were excluded from the study. Patients who died during the follow-up tenure or for whom complete follow-up data set was unavailable were also later excluded.

Patient Data collection

The following data were collected: Demographic and physical information, maternal medical history, relevant past medical history, coexisting diseases, diagnostic laboratory parameters and clinical/biochemical test results. The case notes, charts, investigation results and treatment records of these patients were retrospectively reviewed and statistically analysed by a verified statistician.

Anti-viral treatment and Follow-up

All neonates with active HCMV infection were administered with intravenous gancyclovir (6mg/Kg/12hours for 6 weeks).

Follow-up of each patient was done for 6 months. Data were collected at the end of 3 months and 6 months from the date of admission at hospital.

Supplementary Appendix 2 Sample collection

EDTA (Ethylenediaminetetraacetic acid) anti coagulated peripheral blood (5-10 mL) was collected from patients in vacutainer tubes, processed immediately and serum was separated from the whole blood by centrifugation (1000x g for 10 min). Serum was quickly frozen at

*−*80°C and stored until processed. DNA isolation from serum

DNA was isolated from the blood serum using QIamp DNA blood Mini Kit (Qiagen Inc., Hilden, Germany; 51106) as per manufacturer’s protocol and remaining serum was kept at - 80°C. DNA concentration was measured by measuring OD values using spectrophotometer.

HCMV Qualitative PCR for virus detection

We designed the sequence of primers in the UL 83 and gB regions of HCMV genome using primer 3 online software and using HCMV AD169 strain genome as reference. The primers were obtained from Eurofins Genomics India Pvt. Ltd. The forward and reverse primers for UL 83, 5'-GGG ACA CAA CAC CGT AAA GC-3' and 5'-GTC AGC GTT CGT GTT TCC

CA-3' respectively. The forward and reverse primers for UL 55 (gB) were 5'- GGTCTTCAAGGAACTCAGCAAGA-3' and 5'-CGGCAATCGGTTTGTTGTAAA-3'

respectively. For each 25 μL PCR reaction mixture, 12.5 μL of 2X master mix (Emerald Amp GT PCR Master Mix, TAKARA) , 1.5 ul of each forward and reverse primer (10um Conc.), 6.6 μL sterile water and 2 μL of DNA samples were used. Thermal cycling was initiated with a denaturation step of 95°C for 5 mins. It was followed by 35 cycles of 95°C for 45 seconds, 57.5°C for 30 seconds, 72°C for 45 seconds and final extension at 72°C for 5 mins.

Real-time PCR quantification of HCMV viral load

A quantitation standard curve was achieved by using six 10-fold serial dilutions of a standard HCMV DNA with known viral load (copies/ml) purchased from ATCC. A conserved partial region of the HCMV UL 75 (gH) gene was amplified in each case and Ct value was measured in a real time PCR instrument (ABI 7500- Applied Biosystems). The forward and reverse primers were as follows: 5'-CGTGGAAGATGACCGAAGAT-3' and 5'- ATCGGCCACACTTTAACCAG-3' respectively. The standard DNA concentration was also calibrated by spectrophotometry at 260 nm. A 25ul of total volume of reaction mixture was made with 5ul of DNA, 12.5ul of 2x master mix containing SYBR green (TB green premix Ex Taq, TAKARA), gH forward/reverse primers and water. The cycling conditions were as follows, denaturation at 94^o^C for 5 mins, followed by 35 cycles of 94°C for 30 seconds, 60°C for 30 seconds, 72°C for 30 seconds and final extension at 72°C for 7 mins.

Cytokine ELISA

Serum Tumor Necrosis Factor alpha (TNF-*α*), Interferon gamma (IFNγ), Interlukin 6 (IL-6), Interlukin 10 (IL10), Interlukin 1 beta (IL1β), Transforming growth factor beta (TGF-β), Interlukin 1 Receptor antagonist (IL1Ra), Interlukin 18 (IL18), Interlukin 8 (IL8) and C- reactive protein (CRP) levels were measured by using enzyme-linked immunosorbent assay (ELISA) technique kits from Abcam biotech co., Cambridge, UK and G-Biosciences, Geno Technology Inc., USA. These assays detected only human cytokines and at very low serum concentrations. ELISA was performed as per manufacturer’s protocol.

Isolation and culture of PBMCs

Peripheral blood mononuclear cells were isolated from peripheral blood samples by Ficoll- Paque density gradient centrifugation using histopaque separation medium (Sigma-Aldrich; Merck KGaA). Isolated mononuclear cells were washed and centrifuged twice with PBS lotion containing 3% fetal bovine serum (FBS) and 10 mM EDTA for 15 min at 400 x g at room temperature. Subsequently, the peripheral blood mononuclear cells were resuspended at a final concentration of 1.0x10^7^ cells/ml. PBMCs from each group were cultured and proliferated separately with RPMI-1640 culture medium containing 10% FBS (both from Thermo Fisher Scientific, Inc.) for 24 h in vitro. Then, the PBMCs were harvested and used for RNA isolation.

RNA isolation and real-time polymerase chain reaction

Total RNA was extracted using TRIzol reagent (Invitrogen; Thermo Fisher Scientific, Inc.) according to the manufacturer's instructions. Primescript 1^st^ strand cDNA synthesis kit, TAKARA was used to convert RNA into cDNA at 42˚C for 60 min following standard procedure. For real-time polymerase chain reaction quantification, 2 µl of cDNA was amplified in a 20-µl standard real time PCR reaction with SYBR green dye (TB Green premix ex taq, TAKARA). The detection of GAPDH transcripts provided an internal control in the real time PCR, standardizing the quantity of input cDNA. The real time expression ratios of mRNAs were determined using the 2^-ΔΔCt^ method for relative quantification. All real time primers were designed in the laboratory using primer-3 software and obtained from IDT technologies INC, India. The reaction parameters and conditions were standardized in the laboratory. All the primers that have been used in this study for real time expression have been listed in Supplementary table 1.

Statistical analysis

Results were expressed as mean ± standard deviation, unless otherwise indicated. Differences between groups were compared by unpaired t-testing and one way analysis of variance (ANOVA) when distributions were normal. Non parametric tests were used if they were not normal. Bonferroni method was used with ANOVA for comparing between individual groups. Binary logistic regression was performed to assess statistical significance among two groups of interest. The level of significance (P value) was set at 5%. All P values were two tailed. All statistical analyses were carried out with SPSS software (version 16.0; SPSS, Inc., Chicago, IL, USA). Post-hoc sample size calculation analysis gave a power value of 74.3%

with a total of 110 subjects and alpha value 0.05. All graphs were prepared using graph pad prism

Gene Sequencing

Sequencing primers were designed using primer 3 software to partially amplify a major conserved region of HCMV UL 73 (gN) and UL100 (gM) genes from 24 clinical samples belonging to group 1 (12 IHC and 12 EHC samples). The forward and reverse primers for gM were as follows: 5'-AAGCTGTCGTGGTGACTGAG-3' and 5'- CTCGCACGTGGATAAGGTGA-3' respectively. The forward and reverse primers for gN were as follows: 5'- TCGGTAGTGGCAGAGAGTTC-3' and 5'- TAGCCTTTGGTGGTGGTTGC-3' respectively. The primers were obtained from Eurofins Genomics India Pvt. Ltd. Thermal cycler was used to amplify the specified region. These crude PCR products were outsourced to Agrigenome Labs Pvt. Ltd for Sanger sequencing. After obtaining the sequences they were analysed and submitted to NCBI gene data bank. (GenBank accession no. MN969448-MN969471 for 24 gM sequences and MN969472- MN969495 for 24 gN sequences).

Supplementary Appendix 3 Bioinformatics analysis Sequence Data retrieval:

The complete nucleotide sequences for HCMV gM and gN genes from 16 different standard reference HCMV strains, were obtained from the National Center for Biotechnological Information (NCBI) (available at <https://www.ncbi.nlm.nih.gov/>). The strains were selected from different geographic locations.

Phylogenetic and Evolutionary Analysis:

We had 24 partial nucleotide sequences corresponding to each of HCMV gM and gN genes amplified from clinically isolated HCMV strains and 16 NCBI reference nucleotide sequences from different HCMV strains. The nucleotide sequences were aligned using MUSCLE and the poorly aligned regions with more than 20% gaps were trimmed using trimAl. Coalescent trees were constructed through Bayesian analysis and the time of the most recent common ancestor (MRCA) for some strains and lineages was calculated using BEAST package V.2.6.0 with Markov Chain Monte Carlo (MCMC) algorithm implemented in it. The XML file was generated in BEAUTi program using gamma parameter of site heterogeneity at 1000000 chain-lengths. Sampling prior and mean clock rate were estimated in Tracer software. iTOL was used to visualize the phylogenetic tree.

Analysis of effective number of codons (ENc) and codon adaptation index (CAI):

The ENc value describes the degree that the codon usage deviates from the random selection and depicts the level of preference for the non-equilibrium use of synonymous codons. The ENc values range from 20 to 61. The ENc value of 20 indicates a higher codon usage bias and the value 61 indicates no bias at all. The ENc value were calculated using the chips

programme available in EMBOSS package ([http://www.bioinformatics.nl/cgi-](http://www.bioinformatics.nl/cgi-bin/emboss/chips) [bin/emboss/chips](http://www.bioinformatics.nl/cgi-bin/emboss/chips)). An analysis of variance (ANOVA) test was performed to determine whether the ENc values were significantly different between the EHC and IHC groups. Codon adaptation index (CAI) is a measure that predicts the highest relative adaptation of the viruses to their potential host. CAI programmer available in EMBOSS package (<http://www.bioinformatics.nl/cgi-bin/emboss/cai>) was used to calculate the CAI. CAI values range from 0 to 1. The reference dataset for *Homo sapiens* were retrieved from the Codon Usage Database (<http://www.kazusa.or.jp/codon/>).

Analysis of mRNA secondary structure:

Viral mRNA structure plays a vital role in shaping the evolutionary dynamics of viruses and modulating their interaction with their host. Conserved local secondary structures of viral mRNAs are associated with their specific functions. Here we analyzed the conserved local secondary structure of the gM and gN gene to understand the structural patterns between the clinical strains belonging to two separate groups i.e HCMV induced IHC and HCMV induced EHC. LocARNA was used to generate the local structural alignment and consensus secondary structure. The highly conserved residues in the consensus structure were highlighted in red. RNAscClust was used for performing structural classification of mRNA between two groups.
